# Correlates of hysterectomy in low- and middle-income countries: a systematic review

**DOI:** 10.7189/jogh.16.04172

**Published:** 2026-06-05

**Authors:** Jesty Saira Varghese, Sapna Desai, Sisir Debnath, Sourabh Bikas Paul, Gita D Mishra

**Affiliations:** 1 University of Queensland – Indian Institute of Technology Delhi (UQ-IITD) Research Academy, Hauz Khas, New Delhi, India; 2 Department of Humanities and Social Sciences, Indian Institute of Technology Delhi, Hauz Khas, New Delhi, India; 3 Australian Women and Girls’ Health Research Centre, School of Public Health, Faculty of Health, Medicine and Behavioural Sciences, The University of Queensland, Brisbane, Queensland, Australia; 4 Population Council Institute, New Delhi, India

## Abstract

**Background:**

Hysterectomies for benign conditions have declined in recent decades in most high-income countries (HICs); however, it remains a common surgical procedure among women of reproductive age in low- and middle-income countries (LMICs). Although an expanding body of evidence has examined the factors associated with hysterectomy in HICs, evidence remains limited in LMICs. Therefore, we systematically reviewed and synthesised the existing evidence on correlates of hysterectomy in LMICs.

**Methods:**

We comprehensively searched PubMed, Embase, CINAHL, Scopus, and Web of Science for eligible studies published up to 28 April 2025. We included quantitative studies with population-based samples that examined the correlates of hysterectomy in LMICs. Findings were synthesised using a narrative synthesis approach.

**Results:**

We included 22 studies in this review. All studies were published from 2016 onwards, and three-quarters were conducted in India. Evidence from multiple LMICs, including India, suggests that older age at survey (in reproductive ages), having health insurance at the time of survey, being currently married or having a partner, and belonging to higher wealth quintiles are associated with an increased likelihood of having had a hysterectomy. Studies from India consistently indicate that women with lower levels of education, those who are employed, residing in rural areas, classified as overweight or obese at the time of survey, and having a higher number of children were more likely to have undergone a hysterectomy. Evidence regarding adolescent age at first birth was limited and inconclusive, while evidence on tubal ligation was mixed across studies from India.

**Conclusions:**

We identified several sociodemographic and reproductive correlates of hysterectomy in LMICs. Prospective and mixed-methods studies, as well as studies examining health system factors, including provider incentives and facility-level variation, are needed to inform targeted interventions in LMIC settings.

**Registration:**

PROSPERO: CRD42022335706.

Hysterectomy, the surgical removal of a woman’s uterus, is commonly performed for benign gynaecological conditions such as uterine fibroids, heavy menstrual bleeding, pelvic organ prolapse, and endometriosis [1–3]. Epidemiologic studies report a decline in the incidence of hysterectomy in most high-income countries (HICs) due to increased access to non-surgical alternatives [4,5]. These studies also show that, in addition to biological factors, social and reproductive characteristics are associated with hysterectomy. These include age, education levels, socioeconomic conditions, age at menarche, number of children, health insurance status, and the preferences of healthcare providers [6–11].

To our knowledge, only one systematic review by Wilson and Mishra in 2016 has synthesised evidence on factors associated with hysterectomy, including age at menarche, level of education, and parity [7]. However, all studies included in this review were from HICs and were limited to these three factors. Evidence suggests that the prevalence of hysterectomy and its correlates can differ substantially between and within countries. For instance, a study from Western Australia reported a higher likelihood of hysterectomy among women with lower socioeconomic status [1]. In contrast, studies from India have found an increased likelihood of hysterectomy among women in the highest wealth quintile, with varying correlates across the states [12]. Differences in socioeconomic characteristics, cultural norms, and health system contexts between HICs and low- and middle-income countries (LMICs) limit the generalizability of findings from the existing meta-analysis to LMIC populations.

Existing evidence suggests that population-based studies on the prevalence and correlates of hysterectomy in LMICs remain limited [13]. Nevertheless, hysterectomy appears to be a first-line treatment for benign conditions among young women in India [14]. For instance, a nationally representative survey from India reported that one-half of the hysterectomies among women aged 15–49 years occurred before 34 years [15]. In rural China, one-quarter of the surgeries were performed in women aged <40 years [13]. Further, hysterectomy is the second most performed surgery among women of reproductive age in Brazil [16]. These patterns are concerning because premenopausal hysterectomy is associated with an increased risk of osteoporosis, stroke, cardiovascular disease, and frailty, potentially leading to women approaching older ages with a burden of chronic health conditions [17–20]. The procedure also has psychological consequences, including depression [21] and feelings of loss of womanhood, particularly in cultural contexts where childbearing is regarded as central to a woman’s identity [13,22]. Therefore, understanding the correlates of hysterectomy in LMICs can help identify women at increased risk of the procedure. This can also highlight gaps in existing research, such as factors that remain understudied in these settings. Therefore, this paper aims to systematically review and synthesise the evidence on correlates of hysterectomy in LMICs.

## METHODS

### Search strategy

We registered this systematic review in the PROSPERO (CRD42022335706) and followed the PRISMA guidelines. We searched five databases – PubMed, Embase, CINAHL, Scopus, and Web of Science – for eligible studies published until 28 April 2025. The search strategy combined descriptions of the outcome (hysterectomy), exposure (correlates), and location (LMICs) and was restricted to English-language studies. LMICs were defined according to the World Bank income classification at the time of the search. To ensure comprehensive retrieval, the search strategy included both MeSH for individual LMIC country names and keyword searches for those country names in the title and abstract fields (Appendix S1 in the **Online Supplementary Document**). We also searched the reference lists of studies to identify additional articles. Publication-type restrictions were not applied in our search strategy. Hence, conference proceedings indexed in the selected databases were eligible for inclusion if they met the inclusion criteria. However, we did not conduct a separate search for grey literature. The screening was performed using Covidence (Veritas Health Innovation, Melbourne, Victoria, Australia).

### Eligibility criteria and data extraction

In the review, we included studies if they reported quantitative information on the correlates of hysterectomy in LMICs. Studies that reported only the prevalence, incidence, or indications for hysterectomy without examining correlates were excluded. We included only population-based studies and excluded hospital-based studies or those involving specific population subgroups. Population-based studies were defined as those drawing random samples from a defined general population, such as nationally representative surveys, community-based surveys, or population cohorts. We excluded hospital-based studies because their sampling frames are restricted to women accessing health facilities, which may introduce selection bias and limit generalisability to the general population. This approach is consistent with the prior systematic review that synthesised existing evidence on the association between age at menarche, parity, level of education, and hysterectomy [7]. One investigator (JSV) searched for studies and screened their titles and abstracts. In cases where the eligibility of studies was unclear, a decision was made after discussing it with all investigators. Two investigators (JSV and SiD/SaD) assessed the full texts of eligible studies for final inclusion and extracted data. Any disagreements were resolved through discussion among all investigators. The following information was extracted from each study: first author, year of publication, title, journal, country, data source, study design, study population, methods, the measure of association, sample size, the prevalence of hysterectomy, outcome/comparator, how the outcome was ascertained, and factors considered.

### Quality assessment

Two authors (JSV and GDM/SBP/SiD/SaD) independently assessed the quality of each study using the Newcastle-Ottawa Quality Assessment Scale and resolved any disagreements through discussion. This scale evaluates studies across three domains: sample selection, comparability of study groups, and ascertainment of the outcome (Appendix S2 in the **Online Supplementary Document**) [23]. For this review, the scale was adapted to assign equal points whether the exposure was ascertained through medical records, validated measurement tools, or self-report. We made this adjustment because this review primarily synthesised sociodemographic and reproductive correlates of hysterectomy for which self-report is considered an appropriate method of exposure ascertainment.

### Data analysis and synthesis

The characteristics of the included studies and the results of quality assessment are presented in tabular format. Due to limited evidence from LMICs other than India and heterogeneity in the categorisation of exposure variables across the studies, a meta-analysis could not be performed. Instead, a narrative synthesis was conducted, and findings were reported following the Synthesis Without Meta-analysis guidelines [24]. Factors investigated in at least three studies using distinct data sets were classified as core factors. Based on the exposures examined, these core factors were further grouped into sociodemographic and reproductive factors. Factors examined in fewer than three studies but considered conceptually relevant were categorised as emerging factors. We extracted only adjusted effect estimates, and in studies reporting multiple models, we included those from the most fully adjusted model in the synthesis. For exposures with multiple categories (*e.g.* age, education, household income, number of children, and age at first birth), the highest category reported in each study was compared with the lowest category for ease of comparison [7].

## RESULTS

### Study selection and quality assessment

**Figure 1:**
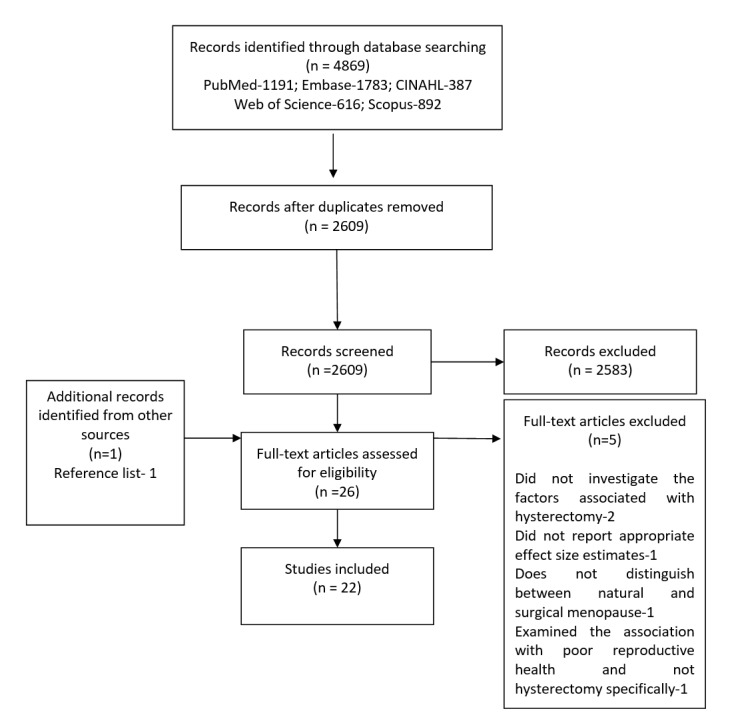
PRISMA flow diagram of the study selection process.

Our search yielded 4869 results. After removing duplicates and screening titles and abstracts, we selected 26 studies for full-text screening, and 22 were included in the review. We identified one additional article through manual searches of the reference lists (**Figure 1**). The total quality scores of the included studies ranged from six to 10 stars (Table S1 and S2 in the **Online Supplementary Document**). Out of the 22 studies, one [13] scored 10 out of 10, 12 [25–36] scored nine out of 10, five [14,37–40] scored eight out of 10, one [41] scored seven out of 10, and two [42,43] scored six out of 10 on the Newcastle-Ottawa scale. A key limitation concerned the method of outcome ascertainment. Hysterectomy status was medically verified in only one study [13] and was self-reported in the other studies. However, ascertainment through medical records is often not feasible in population-based surveys, and previous research has shown that self-reported hysterectomy data are reliable and show high agreement with clinically confirmed cases [44–46].

### Characteristics of the included studies

Half of the studies (n/N = 11/22, 50%) were published between 2016 and 2020, and the remaining were published after 2020. Seventeen studies were from India, and one study each from China, Vietnam, Iran, Brazil, and the Latin America and Caribbean region (**Table 1**). Further, 19 studies employed a cross-sectional design, while three were cohort studies. Among the studies from India, nine used the National Family Health Survey-4 (NFHS-4) as a data source. The sample size of the included studies ranged from 403 to 724 115 respondents. Most studies (n = 13) included women of reproductive age (15–49 years). Six studies recruited broader adult populations, including those aged ≥18 years (n = 2), 35–70 years (n = 2), 30–68 years (n = 1), and 25–69 years (n = 1). Three studies focused exclusively on older women, with age thresholds of 45 years (n = 1), 50 years (n = 1), and 60 years (n = 1).

**Table 1:** Characteristics of the studies included in the systematic review and reported rates of hysterectomy

Study	Country and study period	Data source; study design; study population	Methods; measure of association	Total sample size (% of hysterectomy)	Outcome/comparator	Ascertainment of outcome	Factors examined
Escobar *et al*., 2016 [25]	Barbados, Argentina, Cuba, Mexico, Uruguay, Chile, Brazil. 1999–2000	SABE; cross-sectional; women aged ≥60 years	Multivariable logistic regression analysis; OR	6549 (17.4%); 11% hysterectomy with oophorectomy	Women undergone a hysterectomy *vs.* women not undergone a hysterectomy	Self-reporting by women	Age, marital status, education, household crowding, financial strain, health insurance (at survey), parity, ethnic background, cities
Liu *et al*., 2017 [13]	China, 2009–2011	Questionnaires collected from a subsample of an ongoing oesophageal cancer cohort study; cross-sectional; female residents aged 25–69 years in rural Anyang, China	Univariate and bivariate generalised estimating equation; OR	3328 (3.31)	Women undergone a hysterectomy *vs.* women not undergone a hysterectomy	Ascertained by a gynaecologist	Age, marital status, employment, education, income, cigarette smoking, alcohol consumption, BMI, age at menarche, menstruation regularity, age at first birth, parity, history of pregnancy loss, use of intrauterine contraceptive device, tubal ligation, history of cervicitis, history of vaginitis, history of pelvic inflammation, history of post intercourse bleeding, genital washing before sex
Desai *et al*., 2017 [14]	India, 2010–2012	A mixed-methods, population-based cohort study in Ahmedabad district conducted by the Self-Employed Women’s Association; cohort study; women aged >18 years who have not undergone hysterectomy prior to the study period	Poisson regression, multivariable Poisson regression model; RR	Women surveyed at baseline: 1743; incidence: 20.7/1000 women	Cases of hysterectomy reported over the survey period	Self-reporting by the primary adult respondent	Insurance status (at baseline), location, age at start of follow-up, religion, marital status, number of surviving children, sterilisation history, education, occupation, income, house type, individual latrine, perception of own health
Prusty *et al*., 2018 [26]	India, 2012–2013	DLHS-4; cross-sectional; married women aged 15–49 years in 21 states/UTs	Multivariate logistic regression; OR	316 361 (1.7)	Women undergone a hysterectomy *vs.* women not undergone a hysterectomy	Self-reporting by women	Age, education, caste, religion, place of residence, wealth index, parity, working status, sterilisation, household health insurance (at survey)
Jones *et al*., 2018) [40]	Vietnam, 2007–2008	Retrospective cohort study; women in the northern Vietnamese provinces of Ha Nam, Nam Dinh, Ninh Binh and Thai Binh who had their first Quinacrine sterilisation procedure, last intrauterine device insertion or tubal ligation between 1989 and 1996	Cox proportional hazards regression; HR	21 037 (1.5); incidence: 91 per 100 000 women years of follow-up time	Women undergone a hysterectomy *vs.* women not undergone a hysterectomy	Self-reporting by women	Quinacrine hydrochloride pellet system *vs.* intrauterine devices or tubal ligation for contraception; propensity score- procedure date, date of birth, height, weight, occupation, age at first period, age at first pregnancy, age at first live birth, total pregnancies, total live births, breastfed, years of breastfeeding, exposure to pesticides, exposure to asbestos, exposure to silica, exposure to exhaust
Shekhar *et al*., 2019 [37]	India, 2015–2016	NFHS-4; cross-sectional; women aged 30–49 years	Multivariate binary logistic regression; OR	340 127 (6.1)	Women undergone a hysterectomy *vs.* women not undergone a hysterectomy	Self-reporting by women	Age, education, caste, religion, place of residence, wealth index, BMI (at survey), marital status, age at first cohabitation, parity, region
Desai *et al*., 2019 [12]	India, 2015–2016	NFHS-4; cross-sectional study; women aged 15–49 years	Multivariate binary logistic regression analysis; OR	Overall prevalence not reported. Age specific prevalence only reported	Women undergone a hysterectomy *vs.* women not undergone a hysterectomy	Self-reporting by women	Age, education, caste, religion, place of residence, wealth index, parity, sterilisation status
P Geetha *et al*., 2019 [41]	India, 2019	Information collected from a random sample of rural women in Chittoor district in 2019; cross-sectional; women aged 15–50 years	Age-adjusted multivariable binary logistic regression model; prevalence OR	403 (5.2)	Women undergone a hysterectomy *vs.* women not undergone a hysterectomy	Self-reporting by women	Age, education, income, self-rated health, age at menarche, age at first conception, total conceptions
Meher and Sahoo, 2019 [38]	India, 2015–2016	NFHS-4; cross-sectional study; ever-married women aged 15–49 years (excluded gauna not performed)	Multivariate binary logistic regression analysis; OR	540 671 (4.1)	Women undergone a hysterectomy *vs.* women not undergone a hysterectomy	Self-reporting by women	Age, education, caste, religion, place of residence, wealth index, BMI (at survey), age at first cohabitation, parity, region, working status, and sterilisation
Ensor *et al*., 2020 [39]	India, 2015–2016	NFHS-4, HMIS 2019, Census 2011; cross-sectional; women aged 15–49 years in the eight Northeastern states (Arunachal Pradesh, Assam, Manipur, Meghalaya, Mizoram, Nagaland, Sikkim and Tripura)	Multivariate logit analysis; coefficient and t-statistics	98 700 (0.08)	Women undergone a hysterectomy *vs.* women not undergone a hysterectomy	Self-reporting by women	Age, education, parity, caste/tribe, marital status, insurance held (at survey), economic status; number of doctors, other medical staff, beds per 1000 population; distance between clusters of households and main surgical centres
Singh *et al*., 2020 [43]	India, 2015–2016	NFHS-4; cross-sectional; ever-married women aged 15–49 years (excluded gauna not performed)	Multivariate binary logistic regression analysis; OR	527 865 (4.1)	Women undergone a hysterectomy *vs.* women not undergone a hysterectomy	Self-reporting by women	Age, education, caste, religion, place of residence, wealth index, children ever born, region
Singh and Govil, 2021 [27]	India, 2015–2016	NFHS-4; cross-sectional study; ever-married women aged 15–49 years	Multivariate binary logistic regression analysis; OR	699 686 (3.2) all women prevalence	Women undergone a hysterectomy *vs.* women not undergone a hysterectomy	Self-reporting by women	Age, education, caste, religion, place of residence, wealth index, parity, sterilisation, insurance, age at marriage
Mozumdar, 2021 [28]	India, 2015–2016	NFHS-4; cross-sectional study; nonpregnant women aged 30–49 years	Binary logistic regression; OR	316 179 (6.4)	Women who had surgical menopause *vs.* women with either natural menopause or no menopause	Self-reporting by women	Age (in years), education, social group, residence, wealth index, number of children, BMI (at survey), occupation status, region, age at first birth, anaemic status, ever use of contraception
Kumari *et al*., 2022 [29]	India, 2019–2021	NFHS-5; cross-sectional study; women aged 15–49 years	Binary logistic regression; OR	724 115 (3.3)	Women undergone a hysterectomy *vs.* women not undergone a hysterectomy	Self-reporting by women	Age, education, caste, religion, place of residence, wealth index, parity, region, age at first cohabitation, marital status
Rajkumari *et al*., 2022 [42]	India, baseline study: July 2012–May 2013; follow-up study: September 2020–March 2021	A cohort study among ever-married women aged 35–70 years from the Jat community from Palwal district, Haryana; baseline study: July 2012–May 2013; follow-up study: September 2020–March 2021	Logistic regression; OR	Baseline study: 946; endline study: 702; 11.59 per 1000 women-years	Cases of hysterectomy reported over the survey period	Self-reporting by women	Age, educational status, occupation, smoking, alcoholism, age at menarche, age at first conception, age at last conception, history of foetal loss, tubal ligation, BMI (at survey), hip circumference, blood pressure, lipid parameters
Desai *et al*., 2023 [30]	India, 2017–2018	LASI; cross-sectional data from the first wave of the longitudinal study, Women aged ≥45 years	Multivariable logistic regression; OR	35 083 (11.4)	Women undergone a hysterectomy *vs.* women not undergone a hysterectomy	Self-reporting by women	Age, education, caste, religion, place of residence, monthly *per capita* expenditure (quintile), number of children, marital status, BMI category (at survey), ever employed for three or more months, state
Rout *et al*., 2023 [31]	India, 2017–2018	LASI; cross-sectional data from the first wave of the longitudinal study; women aged >18 years	Multivariable logistic regression; OR	38 154 (11.35)	Women undergone a hysterectomy *vs.* women not undergone a hysterectomy	Self-reporting by women	Age, age at marriage (in years), marital status, place of residence, caste, education, occupation, health insurance (ay survey), MPCE quintile, number of children, physical activity, body mass index (at survey)
Singh *et al*., 2024 [32]	India, 2015–2016 and 2019–2021	NFHS-4, NFHS-5; cross-sectional; women aged 15–49 years in Andhra Pradesh, Telangana, and Bihar	Multilevel logistic regression model; OR	80 976 (7.19)	Women undergone a hysterectomy *vs.* women not undergone a hysterectomy	Self-reporting by women	Age, years of schooling, caste/tribe, religion, place of residence, children ever born, wealth index, health insurance (at survey), ever use of family planning
Datta *et al*., 2024 [33]	India, 2019–2021	NFHS-5; cross-sectional; ever-married women aged 20–49 years	Multivariable logistic regression and nonparametric Kaplan-Meier survival function; OR	528 761 (4.3)	Women undergone a hysterectomy *vs.* women not undergone a hysterectomy	Self-reporting by women	Child marriage, adolescent childbirth, age, place of residence, wealth index, education, religion, caste, BMI (at survey), parity, state fixed effects
Afonso *et al*., 2024 [34]	Brazil, 2015–2016	ELSI Brazil; cross-sectional; women aged ≥50 years	χ^2^ test, Poisson regression; prevalence ratios	5293 (17.8)	Women undergone a hysterectomy *vs.* women not undergone a hysterectomy	Self-reporting by women	Age, education, marital status, region of residence, area of residence, current smoker, had already taken a preventive exam for cervical cancer, number of births, hormone treatment, visited doctor in the last 12 months, having a private health plan (at survey)
Moosazadeh *et al*., 2024 [35]	Iran, 2015–2017	TCS; cross-sectional; women aged 35–70 years who reside in Sari, Mazandaran province in Northern Iran	Multiple logistic regression; OR	6103 (9.7)	Women undergone a hysterectomy *vs.* women not undergone a hysterectomy	Self-reporting by women	Age, education level, socioeconomic status, area of residence, has a job, tubectomy, BMI (at survey), physical activity level, age at first pregnancy, number of pregnancies (gravida)
Singh *et al*., 2024 [36]	India, 2015–2016	NFHS-4; cross-sectional study; women aged 15–49 years from Andhra Pradesh who are not covered under any health insurance plan except Arogyasri, and those who did not undergo a hysterectomy before the launch of the Arogyasri scheme	Propensity score matching; average effects of treatment on treated	2768 (35.08)	Women undergone a hysterectomy *vs.* women not undergone a hysterectomy	Self-reporting by women	Coverage in Andhra Pradesh’s state-specific public-funded health insurance scheme at survey (Aarogyasri scheme); propensity score estimation- age, education, obstetric history, undergone sterilisation, parity, wealth index, social category, religion, rural/urban residence, presence of non-communicable diseases

BMI – body mass index, DLHS – District Level Household and Facility Survey, ELSI – Longitudinal Study of Ageing, HMIS – Health Management Information System, HR – hazard ratio, LASI – Longitudinal Ageing Study in India, MPCE – Monthly Per Capita Consumption Expenditure, NFHS – National Family Health Survey, OR – odds ratio, RR – rate ratio, SABE – Health, Well-being, and Aging Study, TCS – Tabari Cohort Study, UTs – union territories

The prevalence of hysterectomy varied widely across settings. An analysis of nationally representative cross-sectional data from India (NFHS-4) reported that, among women aged 40–49 years, the proportion who had undergone hysterectomy ranged from 22.4% in Andhra Pradesh to 3% in Assam [12]. The median age at hysterectomy among these women was 37 years [12]. Outside India, reported prevalence was 3.3% in rural China (women aged 25–69 years; mean age of 44 years) [13], 9.7% in Iran (women aged 35–70 years) [35], 17.4% in the Latin America and Caribbean region (women aged ≥60 years, median age of 47 years) [25], and 17.8% in Brazil (women aged ≥50 years) [34] (**Table 1**).

In India, national survey data among women of reproductive age indicated that over half of those who had undergone hysterectomy reported excessive menstrual bleeding/pain as a reason for the procedure, followed by fibroids/cysts (20–25%) [29,37]. Similar patterns were observed among older women, although uterine prolapse was also frequently reported [30]. Uterine fibroids/myomas were the leading indication in Vietnam (90%) [40], rural China (71%) [13], and Brazil (11.1%) [34], while dysfunctional or abnormal bleeding accounted for 11% of cases in China [13] and 1.8% in Brazil [34].

### Summary of factors associated with hysterectomy

In three or more studies, 11 factors were examined and classified as core factors. These were further grouped into sociodemographic and reproductive factors. Other conceptually relevant factors examined in fewer than three studies were classified as emerging (Tables S3 and S4 in the **Online Supplementary Document**).

We observed differences in the magnitudes of effect estimates across studies that used the same data source (NFHS-4). These differences may reflect variations in the analytical sample, covariates included in the multivariable models, and analytical approaches. For example, Shekhar *et al*. [37] restricted the sample to women aged 30–49 years, whereas Desai *et al*. [12] included women aged 15–49 years. In addition, although most studies adjusted for sociodemographic characteristics such as age at survey, income, and education, reproductive factors, such as tubal ligation, were included in only a few studies. While most studies used multivariable binary logistic regression, Singh *et al*. [32] employed a multilevel logistic regression model.

### Sociodemographic factors

#### Age

Age was the most frequently examined sociodemographic factor, with 16 cross-sectional studies and one cohort study assessing its association with hysterectomy (Table S3 in the **Online Supplementary Document**). Further, 10 cross-sectional studies from India among women of reproductive age (15–49 years) reported evidence of a positive association between increasing age at survey age and hysterectomy. Similar positive associations were observed in cross-sectional studies from China and Iran among women in the broader adult population (25–69 years and 35–70 years) [13,35]. In contrast, a cross-sectional study conducted in the Latin America and Caribbean region among women aged ≥60 years reported a marginal decrease in the odds of hysterectomy with each additional year of age (adjusted odds ratio (aOR) = 0.97; 95% confidence interval (CI) = 0.96–0.98) [25]. The cohort study conducted among women residing in rural areas in Gujarat, India, which assessed incident hysterectomy, found a lower incidence among women aged ≥55 years at baseline compared with those aged 35–44 years [14].

#### Education

The association between educational level and hysterectomy was assessed in 14 cross-sectional studies. Of the 12 cross-sectional studies from India, nine reported a lower likelihood of hysterectomy among women with the highest level of education compared with those with the lowest. No evidence of an association was observed in the remaining three studies from India, and one study each from the Latin America and Caribbean region and Iran [25,30,31,35,39].

#### Residence

The association between place of residence and hysterectomy was assessed in 11 cross-sectional studies, including 10 from India and one from Iran. Of these, seven studies from India reported a higher likelihood of hysterectomy among women residing in rural areas compared with their urban counterparts. In contrast, two studies from India using data from the Longitudinal Ageing Study in India, in which the sample primarily consisted of women aged ≥45 years, found a higher likelihood of hysterectomy among urban women relative to rural women [30,31]. The study from Iran reported a lower likelihood of hysterectomy among women living in mountainous areas (aOR = 0.57; 95% CI = 0.43–0.75) compared with those residing in urban areas [35].

#### Socioeconomic status (wealth index)

Furthermore, 14 cross-sectional studies and one cohort study examined the association between socioeconomic status and hysterectomy. Of these, 13 were conducted in India and one each in the Latin America and Caribbean region and Iran. Eleven cross-sectional studies from India reported higher odds of hysterectomy among women in higher wealth quintiles than in lower ones. Similarly, a cross-sectional study from Iran showed evidence of higher odds of hysterectomy among women with higher socioeconomic status (aOR = 1.66; 95% CI = 1.13–2.42) compared with those in the lowest category [35]. Across these studies, the wealth index was predominantly based on household assets, although the specific indicators varied between surveys. In contrast, a cohort study from rural areas of Gujarat observed a lower likelihood of hysterectomy among women with higher household income (aOR = 0.12; 95% CI = 0.03–0.45) [14].

#### Employment status

The association between employment status and hysterectomy was examined in six cross-sectional studies, including five from India and one from Iran. Three cross-sectional studies from India reported evidence of a higher odds of hysterectomy among women who were employed, including those engaged in farming, compared with non-working women [26,28,31]. In contrast, the study from Iran reported lower odds of hysterectomy among working women compared with those not employed [35].

#### Marital status

The association between marital status and hysterectomy was examined in six cross-sectional studies and one cohort study. Of these, five were conducted in India and one each in the Latin America and Caribbean region and Brazil. Three studies from India [29,30,39] and the study from Brazil [34] reported evidence of higher odds of hysterectomy among women who were currently married or living with a partner compared with those who were unmarried.

#### Health insurance (at survey)

Furthermore, seven cross-sectional studies assessed the association between health insurance coverage at the time of the survey and hysterectomy, including six from India and one from Brazil. Four studies from India reported evidence of a positive association between having health insurance at the time of survey and a previous history of hysterectomy [26,27,32,36]. In one study that excluded women who reported undergoing hysterectomy prior to the launch of Andhra Pradesh’s state-specific public health insurance scheme, health insurance at the time of survey was associated with a higher probability of having undergone a hysterectomy (average effect of treatment on treated = 0.114, standard error = 0.029) [36]. Similarly, the study from Brazil, which specifically examined private health insurance coverage at the time of survey, reported a positive association between insurance status at survey and history of hysterectomy (adjusted prevalence ratio = 1.02; 95% CI = 1.01–1.03) [34]. In a cohort study among rural women in Gujarat, unadjusted analysis showed no evidence of an association between enrolment in a community-based health insurance scheme for women working in the informal sector and the incidence of hysterectomy (unadjusted rate ratio = 1.01; 95% CI = 0.62–1.64) [14].

#### Body mass index (BMI)

The association between BMI and hysterectomy was examined in seven cross-sectional studies – five from India and one each from China and Iran – and one cohort study from India. Moreover, four cross-sectional studies from India reported evidence of higher odds of having undergone hysterectomy among women classified as obese at the time of survey compared with those who were of normal weight or thin. In contrast, studies from China and Iran report no evidence of an association [13,35].

### Reproductive factors

#### Number of children

The number of children was the most frequently examined reproductive factor, with 16 cross-sectional studies and one cohort study assessing its association with hysterectomy (Table S4 in the **Online Supplementary Document**). Of these, 13 studies were conducted in India, and one each in China, Iran, the Latin America and Caribbean region, and Brazil. Cross-sectional studies from India and Iran generally reported evidence of higher odds of hysterectomy among women with a higher number of children. In contrast, studies from Brazil and the Latin American and Caribbean region reported lower odds of hysterectomy among women with more than three children compared with those with up to three children, whereas a cross-sectional study from China reported no evidence of an association [13,25,34].

#### Tubal ligation

The association between tubal ligation and hysterectomy was assessed in five cross-sectional studies and five cohort studies. Three cross-sectional studies from India based on NFHS-4 data reported evidence of an inverse association between prior tubal ligation and hysterectomy. In contrast, an Indian study using District Level Household and Facility Survey-4 data [26] observed higher odds of hysterectomy among women who had undergone tubal ligation. Similarly, the cross-sectional study from Iran reported a positive association between a history of tubal ligation and hysterectomy [35].

#### Age at first birth

The association between age at first birth and hysterectomy was examined in four cross-sectional studies – three from India and one from Iran – and one cohort study from India. Further, two studies from India reported evidence of a higher odds of hysterectomy among women whose first birth occurred at or before 19 years of age compared with those who had their first birth at later ages [28,33]. The remaining two studies from India [41,42] and a cross-sectional study from Iran reported no evidence of an association [35].

#### Emerging factors

A cross-sectional study from China reported that women who had ever experienced a foetal loss had higher odds of hysterectomy than those who had not (aOR = 1.51; 95% CI = 1.02–2.23) [13]. In addition, a cohort study from India found that women who attained menarche at age ≥16 years had lower odds of hysterectomy compared with those who attained menarche at 13–15 years [42].

Beyond sociodemographic and reproductive characteristics, one study conducted in the Northeast region of India examined health system factors associated with hysterectomy [39]. Greater travel time to the main surgical facility was inversely associated with hysterectomy (coefficient = –0.001, t-statistic (t) = –2.340). However, no associations were observed between the capacity of health facilities, including doctors per 1000 population (coefficient = –0.043, *t* = –1.670), other medical staff per 1000 population (coefficient = 0.011, *t* = 0.990), beds per 1000 population (coefficient = 0.005, *t* = 0.720), and hysterectomy.

## DISCUSSION

This systematic review is, to our knowledge, the first study to offer a comprehensive overview of the current evidence on the correlates of hysterectomy in LMICs. We observed that research on the correlates of hysterectomy remains limited in LMICs, with all included studies published from 2016 onwards, and more than three-quarters of them from India. Evidence from multiple LMICs, including India, suggests that older age at survey (in reproductive ages), having health insurance at the time of survey, being currently married or having a partner, and belonging to higher wealth quintiles are associated with an increased likelihood of having had a hysterectomy. Studies from India consistently indicate that women with lower levels of education, those who are employed, residing in rural areas, classified as overweight or obese at the time of survey, and having a higher number of children were more likely to have undergone a hysterectomy. Evidence regarding adolescent age at first birth was limited and inconclusive, while evidence on tubal ligation was mixed across studies from India.

### Sociodemographic factors

Cross-sectional studies conducted among women predominantly in their reproductive years reported a positive association between age at survey and having ever undergone a hysterectomy. In contrast, a cross-sectional study among women aged ≥60 years showed an inverse association with age at survey [25]. As this analysis was based on ‘ever had hysterectomy,’ the negative association observed in older ages likely reflects cohort or survivor effects rather than a protective effect of advancing age.

Only one cohort study from India assessed incident hysterectomy and found a lower incidence among women aged ≥55 years at baseline compared with those aged 35–44 years [14]. A similar pattern has been reported in HICs, where the incidence of hysterectomy rises with age until 40–50 years, at which point the incidence peaks [4]. Evidence from North Carolina in the USA further suggests that this peak among women aged 40–50 years is primarily driven by a history of uterine leiomyoma, a commonly reported indication for hysterectomy in the studies included in our review [47].

An inverse association between education and hysterectomy was consistently observed in studies from India, aligning with the findings of the systematic review by Wilson and Mishra [7]. Lower education levels may limit women's awareness of reproductive health conditions and available alternative treatments to hysterectomy [7,48]. Consequently, women with lower education may delay seeking treatment for minor gynaecological symptoms, which can worsen over time and ultimately result in hysterectomy [49]. In contrast, most studies reported higher odds of hysterectomy among women from higher wealth quintile households, except for one cohort study conducted among low-income women in Gujarat [14]. This pattern contrasts with HICs and the expected association, as education and income are generally correlated. The reasons for this contrast are not well-explained; however, Shekhar *et al*. suggested that women with lower education may undergo hysterectomy primarily for infections or uterus-related problems that become severe due to delayed care-seeking [37]. In contrast, women from higher-income households may undergo the procedure as they can afford it, with 68% of hysterectomies in India performed in private settings [37]. Studies from Iran also show a high out-of-pocket expenditure, indicating that only women from higher-income households can afford the procedure [50,51].

Multiple studies from LMICs identified health insurance status at the time of survey as a correlate of hysterectomy, and studies from India also reported overweight or obesity at the time of survey as an associated factor. However, all included studies were cross-sectional and measured household health insurance coverage and BMI at the time of the survey rather than at the time of surgery, which makes it difficult to interpret the direction of these associations without information on temporality. Further, examining health insurance at the time of survey rather than at the time of surgery may be misleading. On the one hand, health insurance may increase access to and the affordability of surgical procedures, which may influence providers' or women's decisions to undergo a hysterectomy. On the other hand, women may enrol in health insurance after a hysterectomy due to post-surgical health needs. Also, women may have enrolled in public health insurance after a hysterectomy as government programmes have expanded over time, with no correlation to an earlier decision to undergo a hysterectomy. Similarly, overweight or obesity may act both as a risk factor for gynaecological conditions leading to hysterectomy [52–55] as well as a potential consequence of the procedure [56]. Hence, prospective population-based cohort studies are needed to better understand the association between health insurance, BMI, and hysterectomy.

Studies from India reported a positive association between working status and hysterectomy. One study found that women employed in farming have a higher likelihood of undergoing a hysterectomy compared with women who are not employed [28]. Qualitative evidence from the Beed district in Maharashtra – widely discussed in the media for the high rates of hysterectomy among female sugarcane labourers – suggests that women employed in physically demanding occupations may undergo hysterectomy to avoid work disruptions caused by gynaecological morbidities, and may have increased risk of uterine prolapse [57,58]. In contrast, evidence from Iran indicates that unemployed women may delay seeking treatment, potentially increasing the likelihood of hysterectomy at a later stage [35]. The association between marital status and hysterectomy observed in LMICs may be explained by its link with reproductive history, such as the number of children and age at first birth, which are risk factors for hysterectomy.

Studies from India among women in their reproductive ages found a higher odd of hysterectomy among women in rural areas compared to those from urban areas. A similar association between place of residence and hysterectomy was reported in an Australian study, reflecting limited access to alternative treatment options in rural settings [8]. Qualitative studies from India further suggest that both women from rural areas and their health providers often perceive hysterectomy as the most suitable treatment, as it offers a permanent solution for gynaecological morbidities [14,59]. However, studies among older women from India reported higher odds of hysterectomy among urban women. As hysterectomies in women aged above 45 years may have been performed many years earlier, the higher likelihood of hysterectomy observed among rural women in their reproductive ages could also reflect the increased access to surgical procedures in rural areas [30,31].

### Reproductive factors

Studies from India indicated that women with a higher number of children have a greater likelihood of having had a hysterectomy. Having completed their desired family size, these women would be less likely to avoid the surgery to preserve fertility [60]. Qualitative studies from India further highlighted that both health providers and women often perceive the uterus as a dispensable organ after childbearing is complete [14,59]. Moreover, the risk of damage to gynaecological organs increases with each childbirth, contributing to the higher likelihood of hysterectomy [60]. For instance, the likelihood of uterine prolapse- an indication of a hysterectomy, increases with the number of births among women who had a vaginal birth [61].

An inconsistent association between tubal ligation and subsequent hysterectomy was observed in this review, consistent with findings in the literature [12,48,62]. Some studies reported a higher risk of menstrual disorders and gynaecological ailments among women who had undergone tubal ligation [26,63]. Also, qualitative evidence suggests that women with a history of tubal ligation might be more comfortable having a subsequent surgery than others [14]. However, further research is needed to understand the potential biological and social pathways behind this association. Lastly, the association between adolescent age at first birth and hysterectomy reported in a few studies from India might be due to extended lifetime exposure to oestrogen associated with early childbirth, which may influence the development of gynaecological conditions later in life, potentially leading to hysterectomy [60].

### Further research and policy implications

The younger median age at hysterectomy in India (37 years among women aged 40–49) [12] raises significant concern, particularly as cross-sectional evidence indicates a higher likelihood among women with lower levels of education, those residing in rural areas, and those engaged in physically demanding occupations. While the studies included in this review did not report whether non-surgical options were attempted prior to hysterectomy, heavy menstrual bleeding and fibroids were the leading self-reported indications. Effective alternatives such as ablative methods and hormonal treatments (notably levonorgestrel-releasing intrauterine devices) are available for these conditions [64,65]. These treatments have also contributed to the decline in the incidence of hysterectomy in most HICs [4,5]. Hence, facilitating early diagnosis and increased access to conservative treatment options may help reduce hysterectomy among premenopausal women for benign gynaecological conditions where alternatives are available.

Most studies included in this review were from India and primarily conducted among reproductive-age women, using data from the NFHS (the India Demographic and Health Survey (DHS)). The question on hysterectomy was introduced from the fourth NFHS round, which facilitated a vast body of research on hysterectomy in India. Considering the limited research from other LMICs and the current uncertainty surrounding the future of the DHS programme [66], this review underscores the importance of population-based surveys on women's health for generating robust evidence in these settings. Including standardised questions on hysterectomy and treatment pathways before undergoing hysterectomy would facilitate cross-country comparisons and enable the monitoring of patterns and trends over time.

Also, limited studies included in this review reported a higher likelihood of hysterectomy among women who have ever experienced a terminated pregnancy and a younger age at menarche, and an inverse association between greater travel time to reach the main surgical facility and hysterectomy. Further studies are required to examine the association between reproductive factors- such as age at menarche, hormonal contraceptive use, history of terminated pregnancy, age at first birth, as well as the health system factors associated with hysterectomy.

### Limitations

This systematic review has a few limitations. First, in cross-sectional studies, health insurance status and overweight/obesity were measured at the time of the survey, not at the time of hysterectomy, making it difficult to ascertain temporality and the nature of these associations. Second, the database search and title and abstract screening for this review were conducted by a single author. However, full-text screening and quality assessment were performed independently by two authors. In cases where eligibility was unclear, a decision was made after discussion among all investigators. Third, the restriction to English-language publications may have introduced language bias and potentially underrepresented evidence from certain LMIC settings. Fourth, this review was restricted to population-based studies. Although this enhances generalisability, hospital-based studies may have provided information on provider and facility drivers. Fifth, we could not perform a meta-analysis due to differences in the categorisation of exposure variables and the limited number of studies from LMICs other than India. Lastly, we did not conduct a structured search of grey literature sources, which may introduce publication bias if findings from unpublished studies differ systematically from those included in this review.

## CONCLUSIONS

In this review, we identified several sociodemographic and reproductive correlates of hysterectomy in LMICs. However, most of the studies were from India and based on cross-sectional data. Prospective and mixed-methods studies, as well as studies examining health system factors, including provider incentives and facility-level variation, are needed to inform targeted interventions in LMIC settings. Furthermore, although several correlates of hysterectomy appeared consistent across settings, some differed within the LMICs included in this review, highlighting the importance of context-specific research to inform policy and programmes.

## Additional material


Online supplementary document

## References

[1] Spilsbury K Semmens JB Hammond I Bolck A Persistent high rates of hysterectomy in Western Australia: a population-based study of 83 000 procedures over 23 years. BJOG. 2006;113:804–9. 10.1111/j.1471-0528.2006.00962.x 16827764

[2] Yusuf F Leeder S Wilson A Recent estimates of the incidence of hysterectomy in New South Wales and trends over the past 30 years. Aust N Z J Obstet Gynaecol. 2016;56:420–5. 10.1111/ajo.12477 27297684

[3] Nielsen SL Daugbjerg SB Gimbel H Settnes A Steering Committee of Danish Hysterectomy D. Use of vaginal hysterectomy in Denmark: rates, indications and patient characteristics. Acta Obstet Gynecol Scand. 2011;90:978–84. 10.1111/j.1600-0412.2011.01199.x 21623739

[4] Hammer A Rositch A Kahlert J Gravitt P Blaakaer J Søgaard M Global epidemiology of hysterectomy: possible impact on gynecological cancer rates. Am J Obstet Gynecol. 2015;213:23–9. 10.1016/j.ajog.2015.02.019 25724402

[5] Wilson LF Pandeya N Mishra GD Hysterectomy trends in Australia, 2000-2001 to 2013-2014: joinpoint regression analysis. Acta Obstet Gynecol Scand. 2017;96:1170–9. 10.1111/aogs.13182 28627047

[6] Hanstede MM Burger MJ Timmermans A Burger MP Regional and temporal variation in hysterectomy rates and surgical routes for benign diseases in the Netherlands. Acta Obstet Gynecol Scand. 2012;91:220–5. 10.1111/j.1600-0412.2011.01309.x 22043840

[7] Wilson LF Mishra GD Age at Menarche, Level of Education, Parity and the Risk of Hysterectomy: A Systematic Review and Meta-Analyses of Population-Based Observational Studies. PLoS One. 2016;11:e0151398. 10.1371/journal.pone.0151398 26963512 PMC4786144

[8] Byles JE Mishra G Schofield M Factors associated with hysterectomy among women in Australia. Health Place. 2000;6:301–8. 10.1016/S1353-8292(00)00011-3 11027955

[9] Gimbel H Ottesen B Tabor A Danish gynecologists’ opinion about hysterectomy on benign indication: results of a survey. Acta Obstet Gynecol Scand. 2002;81:1123–31. 10.1034/j.1600-0412.2002.811205.x 12519108

[10] Bickell NA Earp JA Garrett JM Evans AT Gynecologists’ sex, clinical beliefs, and hysterectomy rates. Am J Public Health. 1994;84:1649–52. 10.2105/AJPH.84.10.1649 7943488 PMC1615070

[11] Sievert LL Murphy L Morrison LA Reza AM Brown DE Age at menopause and determinants of hysterectomy and menopause in a multi-ethnic community: the Hilo Women’s Health Study. Maturitas. 2013;76:334–41. 10.1016/j.maturitas.2013.08.007 24054435 PMC3840033

[12] Desai S Shukla A Nambiar D Ved R Patterns of hysterectomy in India: a national and state-level analysis of the Fourth National Family Health Survey (2015-2016). BJOG. 2019;126:72–80. 10.1111/1471-0528.15858 31309706 PMC6772015

[13] Liu F Pan Y Liang Y Zhang C Deng Q Li X The epidemiological profile of hysterectomy in rural Chinese women: a population-based study. BMJ Open. 2017;7:e015351. 10.1136/bmjopen-2016-015351 28667216 PMC5734410

[14] Desai S Campbell OM Sinha T Mahal A Cousens S Incidence and determinants of hysterectomy in a low-income setting in Gujarat, India. Health Policy Plan. 2017;32:68–78. 10.1093/heapol/czw099 27497139 PMC5886266

[15] International Institute for Population Sciences (IIPS) and ICF. National Family Health Survey (NFHS-5), 2019-21. 2021. Available: https://mics.unicef.org/node/2951. Accessed: 26 May 2026.

[16] Augusto KL Brilhante AVM Modesto GCD Saboia DM Rocha CFC Karbage SAL Costs and mortality rates of surgical approaches to hysterectomy in Brazil. Rev Saude Publica. 2018;52:25. 10.11606/S1518-8787.2018052000129 29561962 PMC6257415

[17] Madueke-Laveaux OS Elsharoud A Al-Hendy A What We Know about the Long-Term Risks of Hysterectomy for Benign Indication-A Systematic Review. J Clin Med. 2021;10:5335. 10.3390/jcm10225335 34830617 PMC8622061

[18] Choi HG Jung YJ Lee SW Increased risk of osteoporosis with hysterectomy: A longitudinal follow-up study using a national sample cohort. Am J Obstet Gynecol. 2019;220:573e1–13. 10.1016/j.ajog.2019.02.018 30768935

[19] Yeh JS Cheng HM Hsu PF Sung SH Liu WL Fang HL Hysterectomy in young women associates with higher risk of stroke: a nationwide cohort study. Int J Cardiol. 2013;168:2616–21. 10.1016/j.ijcard.2013.03.042 23587399

[20] Ingelsson E Lundholm C Johansson ALV Altman D Hysterectomy and risk of cardiovascular disease: a population-based cohort study. Eur Heart J. 2011;32:745–50. 10.1093/eurheartj/ehq477 21186237

[21] Choi HG Rhim CC Yoon JY Lee SW Association between hysterectomy and depression: a longitudinal follow-up study using a national sample cohort. Menopause. 2020;27:543–9. 10.1097/GME.0000000000001505 32049924

[22] Marván ML Islas M Vela L Chrisler JC Warren EA Stereotypes of women in different stages of their reproductive life: data from Mexico and the United States. Health Care Women Int. 2008;29:673–87. 10.1080/07399330802188982 18663628

[23] Wells G, Shea B, O’Connell D, Peterson J, Welch V, Losos M, et al. The Newcastle–Ottawa Scale (NOS) for assessing the quality of nonrandomized studies in meta-analyses. Available: https://www.ohri.ca/programs/clinical_epidemiology/oxford.asp. Accessed: 26 May 2026.

[24] Campbell M McKenzie JE Sowden A Katikireddi SV Brennan SE Ellis S Synthesis without meta-analysis (SWiM) in systematic reviews: reporting guideline. BMJ. 2020;368:l6890. 10.1136/bmj.l6890 31948937 PMC7190266

[25] Escobar DA Botero AM Cash MG Reyes-Ortiz CA Factors associated with hysterectomy among older women from Latin America and the Caribbean. Women Health. 2016;56:522–39. 10.1080/03630242.2015.1101738 26478957

[26] Prusty RK Choithani C Gupta SD Predictors of hysterectomy among married women 15-49 years in India. Reprod Health. 2018;15:3. 10.1186/s12978-017-0445-8 29304867 PMC5756367

[27] Singh A Govil D Hysterectomy in India: Spatial and multilevel analysis. Womens Health (Lond). 2021;17:17455065211017068. 10.1177/17455065211017068 34096404 PMC8188977

[28] Mozumdar A Prevalence and Associates of Natural Menopause and Surgical Menopause Among Indian Women Aged 30 to 49 Years: An Analysis of the National Family Health Survey. Womens Reprod Health (Phila). 2021;8:203–21. 10.1080/23293691.2021.1973846

[29] Kumari P Kundu J Prevalence, socio-demographic determinants, and self-reported reasons for hysterectomy and choice of hospitalization in India. BMC Womens Health. 2022;22:514. 10.1186/s12905-022-02072-7 36503443 PMC9743745

[30] Desai S Singh RJ Govil D Nambiar D Shukla A Sinha HH Hysterectomy and women’s health in India: evidence from a nationally representative, cross-sectional survey of older women. Womens Midlife Health. 2023;9:1. 10.1186/s40695-022-00084-9 36609516 PMC9825041

[31] Rout D Sinha A Palo SK Kanungo S Pati S Prevalence and determinants of hysterectomy in India. Sci Rep. 2023;13:14569. 10.1038/s41598-023-41863-2 37666936 PMC10477345

[32] Singh SK Chauhan K Tripathi V Key drivers of hysterectomy among women of reproductive age in three states in India: comparative evidence from NFHS-4 and NFHS-5. BMC Womens Health. 2024;24:107. 10.1186/s12905-024-02886-7 38336664 PMC10854047

[33] Datta BK Tiwari A Role of Child Marriage and Adolescent Childbearing on Hysterectomy Among Married Women in India: A Cross-Sectional and Time-to-Event Analysis. BJOG. 2025;132:2042–51. 10.1111/1471-0528.17950 39238474

[34] Afonso LO Beirith VW de Andrade CR Traebert E de Oliveira C Traebert J Prevalence of hysterectomy and associated factors in Brazilian women aged 50 and older: findings from the Brazilian Longitudinal Study of Ageing (ELSI-Brazil). BMC Public Health. 2024;24:1747. 10.1186/s12889-024-19231-0 38951794 PMC11218412

[35] Moosazadeh M Asadi-Aliabadi M Ghasemi Tirtashi M Pejman M Gheibi M Ghadirzadeh E Prevalence of hysterectomy and its determinants in northern Iran: enrollment results of the Tabari cohort study. BMC Womens Health. 2024;24:502. 10.1186/s12905-024-03338-y 39261840 PMC11389442

[36] Singh V Publicly funded health insurance schemes and demand for health services: evidence from an Indian state using a matching estimator approach. Health Econ Policy Law. 2024;19:429–45. 10.1017/S174413312400001X 38433465

[37] Shekhar C Paswan B Singh A Prevalence, sociodemographic determinants and self-reported reasons for hysterectomy in India. Reprod Health. 2019;16:118. 10.1186/s12978-019-0780-z 31375139 PMC6679457

[38] Meher T Sahoo H Regional pattern of hysterectomy among women in India: Evidence from a recent large scale survey. Women Health. 2020;60:585–600. 10.1080/03630242.2019.1687634 31718517

[39] Ensor T Virk A Aruparayil N Factors influencing use of essential surgical services in North-East India: a cross-sectional study of obstetric and gynaecological surgery. BMJ Open. 2020;10:e038470. 10.1136/bmjopen-2020-038470 33093032 PMC7583072

[40] Jones JK Tave A Pezzullo JC Kardia S Lippes J Long-term risk of hysterectomy and ectopic pregnancy among Vietnamese women using the quinacrine hydrochloride pellet system vs. intrauterine devices or tubal ligation for contraception. Eur J Contracept Reprod Health Care. 2018;23:105–15. 10.1080/13625187.2018.1449823 29683010

[41] Geetha P Bharathi T Surendranadha R K., Kodanda R, K. Prevalence and Correlates of Hysterectomy in Rural Women of Chittoor District, Andhra Pradesh. Annals of Women’s Health. 2019;3:1015.

[42] Rajkumari S Chaudhary V Kasaudhan S Saraswathy KN Incidence and determinants of hysterectomy among North Indian women: An 8-year follow-up study. Front Public Health. 2022;10:1065081. 10.3389/fpubh.2022.1065081 36589953 PMC9800844

[43] Singh SK Sharma SK Siddhanta A Major Correlates and Socioeconomic Inequalities in Hysterectomy among Ever-Married Women in India. Indian J Community Med. 2020;45:12–7. 10.4103/ijcm.IJCM_12_19 32029977 PMC6985941

[44] Colditz GA Stampfer MJ Willett WC Stason WB Rosner B Hennekens CH Reproducibility and validity of self-reported menopausal status in a prospective cohort study. Am J Epidemiol. 1987;126:319–25. 10.1093/aje/126.2.319 3605058

[45] Gentry-Maharaj A Taylor H Kalsi J Ryan A Burnell M Sharma A Validity of self-reported hysterectomy: a prospective cohort study within the UK Collaborative Trial of Ovarian Cancer Screening (UKCTOCS). BMJ Open. 2014;4:e004421. 10.1136/bmjopen-2013-004421 24589827 PMC3939665

[46] Green A Purdie D Green L Dick ML Bain C Siskind V Validity of self-reported hysterectomy and tubal sterilisation. The Survey of Women’s Health Study Group. Aust N Z J Public Health. 1997;21:337–40. 10.1111/j.1467-842X.1997.tb01710.x 9270164

[47] Jacobson GF Shaber RE Armstrong MA Hung Y-Y Hysterectomy Rates for Benign Indications. Obstet Gynecol. 2006;107:1278–83. 10.1097/01.AOG.0000210640.86628.ff 16738152

[48] Dharmalingam A Pool I Dickson J Biosocial determinants of hysterectomy in New Zealand. Am J Public Health. 2000;90:1455–8. 10.2105/AJPH.90.9.1455 10983207 PMC1447627

[49] Lin LP Hsieh M Chen SF Wu CL Hsu SW Lin JD Factors related to hysterectomy in women with physical and mobility disabilities. Res Dev Disabil. 2012;33:990–5. 10.1016/j.ridd.2012.01.002 22502822

[50] Mirabedini SA Fazl Hashemi SME Sarabi Asiabar A Rezapour A Azami-Aghdash S Hosseini Amnab H Out-of-pocket and informal payments in Iran’s health care system: A systematic review and meta-analysis. Med J Islam Repub Iran. 2017;31:70. 10.14196/mjiri.31.70 29445699 PMC5804436

[51] Heidarzadeh A Negari Namaghi R Moravveji A Farivar F Naghshpour P Roshan Fekr F Out-of-pocket and catastrophic health expenditure in Iran. Journal of Public Health. 2024;32:413–9. 10.1007/s10389-023-01824-5

[52] Wise LA Palmer JR Spiegelman D Harlow BL Stewart EA Adams-Campbell LL Influence of body size and body fat distribution on risk of uterine leiomyomata in U.S. black women. Epidemiology. 2005;16:346–54. 10.1097/01.ede.0000158742.11877.99 15824551 PMC1847589

[53] Onstad MA Schmandt RE Lu KH Addressing the Role of Obesity in Endometrial Cancer Risk, Prevention, and Treatment. J Clin Oncol. 2016;34:4225–30. 10.1200/JCO.2016.69.4638 27903150 PMC5455320

[54] Templeman C Marshall SF Clarke CA DeLellis Henderson K Largent J Neuhausen S Risk factors for surgically removed fibroids in a large cohort of teachers. Fertil Steril. 2009;92:1436–46. 10.1016/j.fertnstert.2008.08.074 19019355 PMC2765807

[55] Awwad J Sayegh R Yeretzian J Deeb ME Prevalence, risk factors, and predictors of pelvic organ prolapse: a community-based study. Menopause. 2012;19:1235–41. 10.1097/gme.0b013e31826d2d94 23096244

[56] Moorman PG Schildkraut JM Iversen ES Myers ER Gradison M Warren-White N A prospective study of weight gain after premenopausal hysterectomy. J Womens Health (Larchmt). 2009;18:699–708. 10.1089/jwh.2008.1019 19445617 PMC2851125

[57] Chadha N. Constructing the Laboring Body: An Exploration of Early Hysterectomies, Premature Menopause, and Aging Among Women Laborers in Rural India. In: Irudaya Rajan S, editor. Handbook of Aging, Health and Public Policy: Perspectives from Asia. Singapore: Springer Nature; 2022. p. 1-20.

[58] Geidam AD Goje DJ Prevalence and Risk Factors Associated with the Development of Severe Pelvic Organ Prolapse in the University of Maiduguri Teaching Hospital, Nigeria. Archives of Medicine and Health Sciences. 2022;10:32–6. 10.4103/amhs.amhs_199_21

[59] Sardeshpande N Why do young women accept hysterectomy? Findings from a study in Maharashtra, India. Int J Innov Appl Stud. 2014;8:579–85.

[60] Cooper R Hardy R Kuh D Timing of menarche, childbearing and hysterectomy risk. Maturitas. 2008;61:317–22. 10.1016/j.maturitas.2008.09.025 19013032 PMC3500690

[61] Chiaffarino F Chatenoud L Dindelli M Meschia M Buonaguidi A Amicarelli F Reproductive factors, family history, occupation and risk of urogenital prolapse. Eur J Obstet Gynecol Reprod Biol. 1999;82:63–7. 10.1016/S0301-2115(98)00175-4 10192487

[62] Palmer JR Rao RS Adams-Campbell LL Rosenberg L Correlates of hysterectomy among African-American women. Am J Epidemiol. 1999;150:1309–15. 10.1093/oxfordjournals.aje.a009962 10604773

[63] Hillis SD Marchbanks PA Tylor LR Peterson HB Grp UCRSW Higher hysterectomy risk for sterilized than nonsterilized women: Findings from the US Collaborative Review of Sterilization. Obstet Gynecol. 1998;91:241–6. 10.1016/S0029-7844(97)00648-0 9469283

[64] Australian Commission on Safety and Quality in Health Care and Australian Institute of Health and Welfare. The Second Australian Atlas of Healthcare Variation. Sydney, New South Wales, Australia: Australian Commission on Safety and Quality in Health Care; 2017. Available: https://www.safetyandquality.gov.au/sites/default/files/2026-04/Second%20Australian%20Atlas%20of%20Healthcare%20Variation%20%282017%29.pdf. Accessed: 26 May 2026.

[65] Indian Ministry of Health and Family Welfare. Guidelines to prevent unnecessary hysterectomies. New Delhi, India: Ministry of Health and Family Welfare; 2022. Available: https://www.fogsi.org/wp-content/uploads/announcements/Guidelines-and-Reportings-of-Hysterectomies.pdf. Accessed: 26 May 2026.

[66] Khaki JJ Molenaar J Karki S Olal E Straneo M Mosuse MA When health data go dark: the importance of the DHS Program and imagining its future. BMC Med. 2025;23:241. 10.1186/s12916-025-04062-6 40275318 PMC12023666

