# Supplementary material for: Correlates of hysterectomy in low- and middle-income countries: a systematic review
Source: J Glob Health. 2026 Jun 5;16:04172. doi: 10.7189/jogh.16.04172 (PMC13237810; doi:10.7189/jogh.16.04172)
Supplement: Online supplementary document [file jogh-16-04172-s001.pdf]

Supplement to: Cite as: Varghese JS, Desai S, Debnath S, Paul SB, Mishra GD. Correlates of hysterectomy in low- and middle-income countries: a systematic review. J Glob Health. 2026;16: 04172

Supplementary materials

File S1- Search strategy

PubMed

(("Hysterectomy"[Mesh] OR Hysterectom*[tiab] OR “Uterus removal”[tiab] OR “Removal of uterus”[tiab])

AND

("Sociodemographic Factors"[Mesh] OR "Social Determinants of Health"[Mesh] OR “Causality”[Mesh] OR “Epidemiologic Factors”[Mesh] OR “Risk Factors”[Mesh] OR Epidemiolog*[tiab] OR “Risk factor*”[tiab] OR Determinant*[tiab] OR Factor* [tiab] OR Correlate*[tiab] OR Explanatory*[tiab] OR Predictor*[tiab] OR “Predisposing factor*”[tiab] OR “Contributing factor*”[tiab] OR Associat*[tiab] OR Likel*[tiab])

AND

("Developing Countries"[Mesh] OR “Low and middle income country”[tiab] OR LMIC*[tiab] OR “Low-income country” [tiab] OR “Low income country” [tiab] OR “Middle-income country” [tiab] OR “Middle income country” [tiab] OR “Developing country” [tiab] OR “less developed country”[tiab] OR “under developed country”[tiab] OR “Afghanistan”[Mesh] OR Afghanistan[tiab] OR “Angola”[Mesh] OR Angola[tiab] OR “Algeria”[Mesh] OR Algeria[tiab] OR “Albania”[Mesh] OR Albania[tiab] OR “Argentina”[Mesh] OR Argentina[tiab] OR “Armenia”[Mesh] OR Armenia[tiab] OR “Azerbaijan”[Mesh] OR Azerbaijan[tiab] OR “Burkina Faso”[Mesh] OR (Burkina[tiab] AND Faso[tiab]) OR “Burundi”[Mesh] OR Burundi[tiab] OR “Bangladesh”[Mesh] OR Bangladesh[tiab] OR “Benin”[Mesh] OR Benin[tiab] OR “Bhutan”[Mesh] OR Bhutan[tiab] OR “Bolivia”[Mesh] OR Bolivia[tiab] OR "Republic of Belarus"[Mesh] OR Belarus[tiab] OR “Belize”[Mesh] OR Belize[tiab] OR “Bosnia and Herzegovina”[Mesh] OR (Bosnia[tiab] AND Herzegovina[tiab]) OR “Botswana” [Mesh] OR Botswana [tiab] OR “Brazil” [Mesh] OR Brazil[tiab] OR “Central African Republic”[Mesh] OR “Central African Republic”[tiab] OR “Chad”[Mesh] OR Chad[tiab] OR “Congo”[Mesh] OR Congo[tiab] OR “Cabo Verde”[Mesh] OR “Cabo Verde*”[tiab] OR “Cambodia”[Mesh] OR Cambodia[tiab] OR “Cameroon”[Mesh] OR Cameroon[tiab] OR “Comoros”[Mesh] OR Comoros[tiab] OR “Cote d’Ivoire”[Mesh] OR Cote*[tiab] OR “China”[Mesh] OR China[tiab] OR “Colombia”[Mesh] OR Colombia[tiab] OR “Costa Rica”[Mesh] OR (Costa[tiab] AND Rica[tiab]) OR “Cuba”[Mesh] OR Cuba[tiab] OR “Djibouti”[Mesh] OR Djibouti[tiab] OR “Dominica”[Mesh] OR Dominica[tiab] OR “Dominican Republic”[Mesh] OR “Eritrea”[Mesh] OR Eritrea[tiab] OR “Ethiopia”[Mesh] OR Ethiopia[tiab] OR “Egypt”[Mesh] OR Egypt[tiab] OR “Eswatini”[Mesh] OR Eswatini[tiab] OR “El Salvador”[Mesh] OR “El Salvador*”[tiab] OR “Equatorial Guinea”[tiab] OR “Ecuador”[Mesh] OR Ecuador[tiab] OR “Fiji”[Mesh] OR Fiji[tiab] OR “Gambia”[Mesh] OR Gambia[tiab] OR “Guinea”[Mesh] OR Guinea[tiab] OR “Ghana”[Mesh] OR Ghana[tiab] OR “Gabon”[Mesh] OR Gabon[tiab] OR “Georgia”[Mesh] OR Georgia[tiab] OR “Grenada”[Mesh] OR Grenada[tiab] OR “Guatemala”[Mesh] OR Guatemala[tiab] OR “Haiti”[Mesh] OR Haiti[tiab] OR “Honduras”[Mesh] OR Honduras[tiab] OR “India”[Mesh] OR India[tiab] OR “Indonesia”[Mesh] OR Indonesia[tiab] OR "Iran"[Mesh] OR Iran[tiab] OR “Iraq”[Mesh] OR Iraq[tiab] OR (West[tiab] AND Bank[tiab]) OR Gaza[tiab] OR “Jordan”[Mesh] OR Jordan[tiab] OR “Jamaica”[Mesh] OR Jamaica[tiab] OR “Democratic People’s Republic of Korea”[Mesh] OR “Democratic People’s Republic of Korea”[tiab] OR “North Korea”[tiab]

OR “Kenya”[Mesh] OR Kenya[tiab] OR Kiribati[tiab] OR "Kyrgyzstan"[Mesh] OR Kyrgyz[tiab] OR “Kazakhstan”[Mesh] OR Kazakhstan[tiab] OR “Kosovo”[Mesh] OR Kosovo[tiab] OR “Liberia”[Mesh] OR Liberia[tiab] OR “Lao PDR”[tiab] OR “Lebanon”[Mesh] OR Lebanon[tiab] OR “Lesotho”[Mesh] OR Lesotho[tiab] OR “Libya”[Mesh] OR Libya[tiab] OR “Madagascar”[Mesh] OR Madagascar[tiab] OR “Malawi”[Mesh] OR Malawi[tiab] OR “Mali”[Mesh] OR Mali[tiab] OR “Mozambique”[Mesh] OR Mozambique[tiab] OR “Mauritania”[Mesh] OR Mauritania[tiab] OR “Micronesia”[Mesh] OR Micronesia[tiab] OR “Mongolia”[Mesh] OR Mongolia[tiab] OR “Morocco”[Mesh] OR Morocco[tiab] OR “Myanmar”[Mesh] OR Myanmar[tiab] OR “Malaysia”[Mesh] OR Malaysia[tiab] OR “Maldives”[Mesh] OR Maldives[tiab] OR “Marshall Islands”[tiab] OR “Mauritius”[Mesh] OR Mauritius[tiab] OR “Mexico”[Mesh] OR Mexico[tiab] OR “Moldova”[Mesh] OR Moldova[tiab] OR “Montenegro”[Mesh] OR Montenegro[tiab] OR “Niger”[Mesh] OR Niger[tiab] OR “Nepal”[Mesh] OR Nepal[tiab] OR “Nicaragua”[Mesh] OR Nicaragua[tiab] OR “Nigeria”[Mesh] OR Nigeria[tiab] OR “Namibia”[Mesh] OR Namibia[tiab] OR “Republic of North Macedonia”[Mesh] OR Macedonia[tiab] OR “Pakistan”[Mesh] OR Pakistan[tiab] OR “Papua New Guinea”[Mesh] OR “Philippines”[Mesh] OR Philippines[tiab] OR “Paraguay”[Mesh] OR Paraguay[tiab] OR “Peru”[Mesh] OR Peru[tiab] OR “Rwanda”[Mesh] OR Rwanda[tiab] OR “Sierra Leone”[Mesh] OR Sierra[tiab] OR “Somalia”[Mesh] OR Somalia[tiab] OR “South Sudan”[Mesh] OR Sudan[tiab] OR “Sudan”[Mesh] OR “Syria”[Mesh] OR Syria[tiab] OR “Samoa”[Mesh] OR Samoa[tiab] OR "Sao Tome and Principe"[Mesh] OR “Sao Tome and Principe”[tiab] OR “Senegal”[Mesh] OR Senegal[tiab] OR “Melanesia”[Mesh] OR “Solomon Islands”[tiab] OR “Sri Lanka”[Mesh] OR “Sri Lanka”[tiab] OR “Serbia”[Mesh] OR Serbia[tiab] OR “South Africa”[Mesh] OR “South Africa*”[tiab] OR “Saint Lucia”[Mesh] OR “Saint Lucia”[tiab] OR “Saint Vincent and the Grenadines”[Mesh] OR “Saint Vincent and the Grenadines”[tiab] OR “Suriname”[Mesh] OR Suriname[tiab] OR “Togo”[Mesh] OR Togo[tiab] OR “Tanzania”[Mesh] OR Tanzania[tiab] OR “Tajikistan”[Mesh] OR Tajikistan[tiab] OR “Timor-Leste”[Mesh] OR Timor[tiab] OR “Tunisia”[Mesh] OR Tunisia[tiab] OR “Thailand”[Mesh] OR Thailand[tiab] OR “Tonga”[Mesh] OR Tonga[tiab] OR “Turkey”[Mesh] OR Turkey[tiab] OR “Turkmenistan”[Mesh] OR Turkmenistan[tiab] OR Tuvalu[tiab] OR “Uganda”[Mesh] OR Uganda[tiab] OR “Ukraine”[Mesh] OR Ukraine[tiab] OR “Uzbekistan”[Mesh] OR Uzbekistan[tiab] OR “Vanuatu”[Mesh] OR Vanuatu[tiab] OR “Vietnam”[Mesh] OR Vietnam[tiab] OR Gaza[tiab] OR “Yemen”[Mesh] OR Yemen[tiab] OR “Zambia”[Mesh] OR Zambia[tiab] OR “Zimbabwe”[Mesh] OR Zimbabwe[tiab]))

AND (english[Filter])

<u>Embase</u>

((hysterectomy/exp/mj OR Hysterectom*:ti,ab OR 'Uterus removal:ti,ab' OR 'Removal of uterus:ti,ab')

AND ('sociodemographics'/exp OR 'socioeconomic parameters'/exp OR 'social determinants of health'/exp OR causality/exp OR epidemiology/exp OR 'risk factor/exp' OR 'predictor variable'/exp OR Epidemiolog*:ti,ab OR 'Risk factor*:ti,ab' OR Determinant*:ti,ab OR Factor*:ti,ab OR Correlate*:ti,ab OR Explanatory*:ti,ab OR Predictor*:ti,ab OR Likel*:ti,ab OR Associat*:ti,ab OR 'Predisposing factor*:ti,ab' OR 'Contributing factor*:ti,ab')

AND ('developing country'/exp OR 'low and middle income country'/exp OR 'Low and middle income countr*:ti,ab' OR LMIC*:ti,ab OR 'Low-income countr*:ti,ab' OR 'Low income countr*:ti,ab' OR 'Middle-income

countr*:ti,ab' OR 'Middle income countr*:ti,ab' OR 'Developing countr*:ti,ab' OR 'less developed countr*:ti,ab' OR 'under developed countr*:ti,ab' OR Afghanistan:ti,ab OR Angola:ti,ab OR Algeria:ti,ab OR Albania:ti,ab OR Argentina:ti,ab OR Armenia:ti,ab OR Azerbaijan:ti,ab OR 'Burkina Faso:ti,ab' OR Burundi:ti,ab OR Bangladesh:ti,ab OR Benin:ti,ab OR Bhutan:ti,ab OR Bolivia:ti,ab OR Belarus:ti,ab OR Belize:ti,ab OR 'Bosnia and Herzegovina:ti,ab' OR Botswana:ti,ab OR Brazil:ti,ab OR 'Central African Republic:ti,ab' OR Chad:ti,ab OR Congo:ti,ab OR 'Cabo Verde*:ti,ab' OR Cambodia:ti,ab OR Cameroon:ti,ab OR Comoros:ti,ab OR Cote*:ti,ab OR China:ti,ab OR Colombia:ti,ab OR 'Costa Rica:ti,ab' OR Cuba:ti,ab OR Djibouti:ti,ab OR Dominica*:ti,ab OR Eritrea:ti,ab OR Ethiopia:ti,ab OR Egypt:ti,ab OR Eswatini:ti,ab OR 'El Salvador:ti,ab' OR 'Equatorial Guinea:ti,ab' OR Ecuador:ti,ab OR Fiji:ti,ab OR Gambia:ti,ab OR 'Guinea-Bissau:ti,ab' OR Ghana:ti,ab OR Gabon:ti,ab OR Georgia:ti,ab OR Grenada:ti,ab OR Guatemala:ti,ab OR Haiti:ti,ab OR Honduras:ti,ab OR India:ti,ab OR Indonesia:ti,ab OR Iran:ti,ab OR Iraq:ti,ab OR Jordan:ti,ab OR Jamaica:ti,ab OR 'North Korea:ti,ab' OR Kenya:ti,ab OR Kiribati:ti,ab OR Kyrgyz*:ti,ab OR Kazakhstan:ti,ab OR Kosovo:ti,ab OR Liberia:ti,ab OR 'Lao PDR:ti,ab' OR Lebanon:ti,ab OR Lesotho:ti,ab OR Libya:ti,ab OR Madagascar:ti,ab OR Malawi:ti,ab OR Mali:ti,ab OR Mozambique:ti,ab OR Mauritania:ti,ab OR Micronesia:ti,ab OR Mongolia:ti,ab OR Morocco:ti,ab OR Myanmar:ti,ab OR Malaysia:ti,ab OR Maldives:ti,ab OR 'Marshall Islands:ti,ab' OR Mauritius:ti,ab OR Mexico:ti,ab OR Moldova:ti,ab OR Montenegro:ti,ab OR Niger:ti,ab OR Nepal:ti,ab OR Nicaragua:ti,ab OR Nigeria:ti,ab OR Namibia:ti,ab OR Macedonia:ti,ab OR Pakistan:ti,ab OR 'Papua New Guinea:ti,ab' OR Philippines:ti,ab OR Paraguay:ti,ab OR Peru:ti,ab OR Rwanda:ti,ab OR 'Sierra Leone:ti,ab' OR Somalia:ti,ab OR Sudan:ti,ab OR Syria:ti,ab OR Samoa:ti,ab OR 'Sao Tome and Principe:ti,ab' OR Senegal:ti,ab OR 'Solomon Islands:ti,ab' OR 'Sri Lanka:ti,ab' OR Serbia:ti,ab OR 'South Africa:ti,ab' OR 'Saint Lucia:ti,ab' OR 'Saint Vincent and the Grenadines:ti,ab' OR Suriname:ti,ab OR Togo:ti,ab OR Tanzania:ti,ab OR Tajikistan:ti,ab OR 'Timor-Leste:ti,ab' OR Tunisia:ti,ab OR Thailand:ti,ab OR Tonga:ti,ab OR Turkey:ti,ab OR Turkmenistan:ti,ab OR Tuvalu:ti,ab OR Uganda:ti,ab OR Ukraine:ti,ab OR Uzbekistan:ti,ab OR Vanuatu:ti,ab OR Vietnam:ti,ab OR 'West Bank:ti,ab' OR Gaza:ti,ab OR Yemen:ti,ab OR Zambia:ti,ab OR Zimbabwe:ti,ab)) AND [English]/lim

CINAHL

((MH "Hysterectomy+" OR TI Hysterectom* OR AB Hysterectom* OR TI "Uterus removal" OR AB "Uterus removal" OR TI "Removal of uterus" OR AB "Removal of uterus")

AND

(MH "Socioeconomic Factors+" OR MH “Causality+” OR MH "Epidemiology+" OR MH "Risk Factors+" OR MH "Social Determinants of Health+" OR MH “Sociodemographic Factors+” OR TI Epidemiolog* OR AB Epidemiolog* OR TI “Risk factor*” OR AB “Risk factor*” OR TI Determinant* OR AB Determinant* OR TI Factor* OR AB Factor* OR TI Correlate* OR AB Correlate* OR TI Explanatory* OR AB Explanatory* OR TI Predictor* OR AB Predictor* OR TI Likel* OR AB Likel* OR TI Associat* OR AB Associat* OR TI “Predisposing factor*” OR AB “Predisposing factor*” OR TI “Contributing factor*” OR AB “Contributing factor*”)

AND

(MH "Developing Countries+" OR MH “Low and Middle Income Countries+” OR TI "Low and middle income countr*" OR AB "Low and middle income countr*" OR TI LMIC* OR AB LMIC* OR TI “Low-income countr*” OR AB “Low-income countr*” OR TI "Low income countr*" OR AB "Low income countr*" OR TI “Middle-income countr*” OR AB “Middle-income countr*” OR TI "Middle income countr*" OR AB "Middle income countr*" OR TI “Developing countr*” OR AB “Developing countr*” OR TI "less developed countr*" OR AB "less developed countr*" OR TI "under developed countr*" OR AB "under developed countr*" OR MH “Afghanistan” OR TI Afghanistan OR AB Afghanistan OR MH “Angola” OR TI Angola OR AB Angola OR MH “Algeria” OR TI Algeria OR AB Algeria OR MH “Albania” OR TI Albania OR AB Albania OR MH “Argentina” OR TI Argentina OR AB Argentina OR MH “Armenia” OR TI Armenia OR AB Armenia OR MH “Azerbaijan” OR TI Azerbaijan OR AB Azerbaijan OR MH "Burkina Faso" OR TI “Burkina Faso” OR AB “Burkina Faso” OR MH “Burundi” OR TI Burundi OR AB Burundi OR MH “Bangladesh” OR TI Bangladesh OR AB Bangladesh OR MH “Benin” OR TI Benin OR AB Benin OR MH “Bhutan” OR TI Bhutan OR AB Bhutan OR MH “Bolivia” OR TI Bolivia OR AB Bolivia OR MH "Byelarus" OR TI Belarus OR AB Belarus OR MH “Belize” OR TI Belize OR AB Belize OR MH "Bosnia-Herzegovina" OR TI Bosnia OR AB Bosnia OR TI Herzegovina OR AB Herzegovina OR MH “Botswana” OR TI Botswana OR AB Botswana OR MH “Brazil” OR TI Brazil OR AB Brazil OR MH "Central African Republic" OR TI "Central African Republic" OR AB "Central African Republic" OR MH “Chad” OR TI Chad OR AB Chad OR MH "Democratic Republic of the Congo" OR TI Congo OR AB Congo OR MH "Cape Verde" OR TI "Cabo Verde*" OR AB "Cabo Verde*" OR MH “Cambodia” OR TI Cambodia OR AB Cambodia OR MH “Cameroon” OR TI Cameroon OR AB Cameroon OR TI Comoros OR AB Comoros OR MH "Cote d’Ivoire" OR TI Cote* OR AB Cote* OR MH China OR TI China OR AB China OR MH “Colombia” OR TI Colombia OR AB Colombia OR MH "Costa Rica" OR TI “Costa Rica” OR AB “Costa Rica” OR MH “Cuba” OR TI Cuba OR AB Cuba OR MH “Djibouti” OR TI Djibouti OR AB Djibouti OR MH “Dominica” OR TI Dominica* OR AB Dominica* OR MH "Dominican Republic" OR MH “Eritrea” OR TI Eritrea OR AB Eritrea OR MH “Ethiopia” OR TI Ethiopia OR AB Ethiopia OR MH “Egypt” OR TI Egypt OR AB Egypt OR TI Eswatini OR AB Eswatini OR MH "El Salvador" OR TI "El Salvador" OR AB "El Salvador" OR MH “Equatorial Guinea” OR TI "Equatorial Guinea" OR AB "Equatorial Guinea" OR MH “Ecuador” OR TI Ecuador OR AB Ecuador OR MH “Melanesia” OR TI Fiji OR AB Fiji OR MH “Gambia” OR TI Gambia OR AB Gambia OR MH “Guinea-Bissau” OR TI Guinea OR AB Guinea OR MH “Guinea” OR MH “Ghana” OR TI Ghana OR AB Ghana OR MH “Gabon” OR TI Gabon OR AB Gabon OR MH “Georgia” OR TI Georgia OR AB Georgia OR TI Grenada OR AB Grenada OR MH “Guatemala” OR TI Guatemala OR AB Guatemala OR MH “Haiti” OR TI Haiti OR AB Haiti OR MH “Honduras” OR TI Honduras OR AB Honduras OR MH “India” OR TI India OR AB India OR MH “Indonesia” OR TI Indonesia OR AB Indonesia OR MH “Iran” OR TI Iran OR AB Iran OR MH “Iraq” OR TI Iraq OR AB Iraq OR MH “Jordan” OR TI Jordan OR AB Jordan OR MH “Jamaica” OR TI Jamaica OR AB Jamaica OR MH "North Korea" OR TI "North Korea" OR AB "North Kerala" OR TI "Democratic People’s Republic of Korea" OR AB "Democratic People’s Republic of Korea" OR MH “Kenya” OR TI Kenya OR AB Kenya OR TI Kiribati OR AB Kiribati OR MH “Kyrgyzstan” OR TI Kyrgyztan OR AB Kyrgyztan OR MH “Kazakhstan” OR TI Kazakhstan OR AB Kazakhstan OR MH “Yugoslavia” OR TI Kosovo OR AB Kosovo OR MH “Liberia” OR TI Liberia OR AB Liberia OR MH “Laos” OR TI "Lao PDR" OR AB "Lao PDR" OR MH “Lebanon” OR TI Lebanon OR AB Lebanon OR MH “Lesotho” OR TI Lesotho OR AB Lesotho OR MH “Libya” OR TI Libya OR AB Libya OR MH “Madagascar” OR TI Madagascar OR AB

Madagascar OR MH “Malawi” OR TI Malawi OR AB Malawi OR MH “Mali” OR TI Mali OR AB Mali OR MH “Mozambique” OR TI Mozambique OR AB Mozambique OR MH “Mauritania” OR TI Mauritania OR AB Mauritania OR MH “Micronesia” OR TI Micronesia OR AB Micronesia OR MH “Mongolia” OR TI Mongolia OR AB Mongolia OR MH “Morocco” OR TI Morocco OR AB Morocco OR MH “Myanmar” OR TI Myanmar OR AB Myanmar OR MH “Malaysia” OR TI Malaysia OR AB Malaysia OR MH “Maldives” OR TI Maldives OR AB Maldives OR TI "Marshall Islands" OR AB "Marshall Islands" OR MH “Indian Ocean Islands” OR TI Mauritius OR AB Mauritius OR MH “Mexico” OR TI Mexico OR AB Mexico OR MH “Moldova” OR TI Moldova OR AB Moldova OR TI Montenegro OR AB Montenegro OR MH “Niger” OR TI Niger OR AB Niger OR MH “Nepal” OR TI Nepal OR AB Nepal OR MH “Nicaragua” OR TI Nicaragua OR AB Nicaragua OR MH “Nigeria” OR TI Nigeria OR AB Nigeria OR MH “Namibia” OR TI Namibia OR AB Namibia OR MH "Macedonia (Republic)” OR TI Macedonia OR AB Macedonia OR MH “Pakistan” OR TI Pakistan OR AB Pakistan OR MH "Papua New Guinea” OR MH “Philippines” OR TI Philippines OR AB Philippines OR MH “Paraguay” OR TI Paraguay OR AB Paraguay OR MH “Peru” OR TI Peru OR AB Peru OR MH “Rwanda” OR TI Rwanda OR AB Rwanda OR MH "Sierra Leone" OR TI Sierra OR AB Sierra OR MH “Somalia” OR TI Somalia OR AB Somalia OR TI Sudan OR AB Sudan OR MH “Sudan” OR MH “Syria” OR TI Syria OR AB Syria OR MH “Samoa” OR TI Samoa OR AB Samoa OR TI "Sao Tome and Principe" OR AB "Sao Tome and Principe" OR MH “Senegal” OR TI Senegal OR AB Senegal OR MH “Melanesia” OR TI "Solomon Islands" OR AB "Solomon Islands" OR MH "Sri Lanka" OR TI "Sri Lanka" OR AB "Sri Lanka" OR MH “Serbia” OR TI Serbia OR AB Serbia OR MH "South Africa" OR TI "South Africa" OR AB "South Africa" OR TI "Saint Lucia" OR AB "Saint Lucia" OR TI "Saint Vincent and the Grenadines" OR AB "Saint Vincent and the Grenadines” OR MH “Suriname” OR TI Suriname OR AB Suriname OR MH “Togo” OR TI Togo OR AB Togo OR MH “Tanzania” OR TI Tanzania OR AB Tanzania OR MH “Tajikistan” OR TI Tajikistan OR AB Tajikistan OR MH “Timor” OR TI Timor OR AB Timor OR MH “Tunisia” OR TI Tunisia OR AB Tunisia OR MH “Thailand” OR TI Thailand OR AB Thailand OR MH “Polynesia” OR TI Tonga OR AB Tonga OR MH “Turkey” OR TI Turkey OR AB Turkey OR MH “Turkmenistan” OR TI Turkmenistan OR AB Turkmenistan OR TI Tuvalu OR AB Tuvalu OR MH “Uganda” OR TI Uganda OR AB Uganda OR MH “Ukraine” OR TI Ukraine OR AB Ukraine OR MH “Uzbekistan” OR TI Uzbekistan OR AB Uzbekistan OR TI Vanuatu OR AB Vanuatu OR MH “Vietnam” OR TI Vietnam OR AB Vietnam OR TI Gaza OR AB Gaza OR T1 “West Bank” OR AB “West Bank” OR MH “Yemen” OR TI Yemen OR AB Yemen OR MH “Zambia” OR TI Zambia OR AB Zambia OR MH “Zimbabwe” OR TI Zimbabwe OR AB Zimbabwe))

Scopus

TITLE-ABS (Hysterectom* OR "Uterus removal" OR "Removal of uterus")

AND

TITLE-ABS ("Sociodemographic Factors" OR "Social Determinants of Health" OR Causality OR "Epidemiologic Factors" OR "Risk Factors" OR Epidemiolog* OR Determinant* OR Factor* OR Correlate* OR Explanatory OR Predictor* OR "Predisposing factor*" OR "Contributing factor*" OR Associat* OR Likel*)

AND

TITLE-ABS ("Developing Countries" OR "Low and middle income countr*" OR LMIC* OR "Low-income countr*" OR "Middle-income countr*" OR "Developing countr*" OR "less developed countr*" OR "under developed countr*" OR Afghanistan OR Angola OR Algeria OR Albania OR Argentina OR Armenia OR Azerbaijan OR "Burkina Faso" OR Burundi OR Bangladesh OR Benin OR Bhutan OR Bolivia OR Belarus OR Belize OR "Bosnia and Herzegovina" OR Botswana OR Brazil OR "Central African Republic" OR Chad OR Congo OR "Cabo Verde" OR Cambodia OR Cameroon OR Comoros OR "Cote d'Ivoire" OR China OR Colombia OR "Costa Rica" OR Cuba OR Djibouti OR Dominica OR "Dominican Republic" OR Eritrea OR Ethiopia OR Egypt OR Eswatini OR "El Salvador" OR "Equatorial Guinea" OR Ecuador OR Fiji OR Gambia OR Guinea OR Ghana OR Gabon OR Georgia OR Grenada OR Guatemala OR Haiti OR Honduras OR India OR Indonesia OR Iran OR Iraq OR Jordan OR Jamaica OR "Democratic People's Republic of Korea" OR "North Korea" OR Kenya OR Kiribati OR Kyrgyzstan OR Kazakhstan OR Kosovo OR Liberia OR "Lao PDR" OR Lebanon OR Lesotho OR Libya OR Madagascar OR Malawi OR Mali OR Mozambique OR Mauritania OR Micronesia OR Mongolia OR Morocco OR Myanmar OR Malaysia OR Maldives OR "Marshall Islands" OR Mauritius OR Mexico OR Moldova OR Montenegro OR Niger OR Nepal OR Nicaragua OR Nigeria OR Namibia OR "Republic of North Macedonia" OR Macedonia OR Pakistan OR "Papua New Guinea" OR Philippines OR Paraguay OR Peru OR Rwanda OR "Sierra Leone" OR Somalia OR "South Sudan" OR Sudan OR Syria OR Samoa OR "Sao Tome and Principe" OR Senegal OR Melanesia OR "Solomon Islands" OR "Sri Lanka" OR Serbia OR "South Africa" OR "Saint Lucia" OR "Saint Vincent and the Grenadines" OR Suriname OR Togo OR Tanzania OR Tajikistan OR Timor-Leste OR Tunisia OR Thailand OR Tonga OR Turkey OR Turkmenistan OR Tuvalu OR Uganda OR Ukraine OR Uzbekistan OR Vanuatu OR Vietnam OR "West Bank" OR Gaza OR Yemen OR Zambia OR Zimbabwe)

Web of Science

(Abstract)

(Hysterectom* OR "Uterus removal" OR "Removal of uterus")

AND

("Sociodemographic Factors" OR "Social Determinants of Health" OR Causality OR "Epidemiologic Factors" OR Epidemiolog* OR "Risk factor*" OR Determinant* OR Factor* OR Correlate* OR Explanatory* OR Predictor* OR "Predisposing factor*" OR "Contributing factor*" OR Associat* OR Likel*)

AND

("Developing Countries" OR "Low and middle income country" OR LMIC* OR "Low-income country" OR "Low income country" OR "Middle-income country" OR "Middle income country" OR "Developing country" OR "less developed country" OR "under developed country" OR Afghanistan OR Angola OR Algeria OR Albania OR Argentina OR Armenia OR Azerbaijan OR "Burkina Faso" OR (Burkina AND Faso) OR Burundi OR Bangladesh OR Benin OR Bhutan OR Bolivia OR "Republic of Belarus" OR Belarus OR Belize OR "Bosnia and Herzegovina" OR (Bosnia AND Herzegovina) OR Botswana OR Brazil OR "Central African Republic" OR Chad OR Congo OR "Cabo Verde" OR "Cabo Verde*" OR Cambodia OR Cameroon OR Comoros OR "Cote d’Ivoire" OR Cote* OR China OR Colombia OR "Costa Rica" OR (Costa AND Rica) OR Cuba OR Djibouti OR Dominica

OR "Dominican Republic" OR Eritrea OR Ethiopia OR Egypt OR Eswatini OR "El Salvador" OR "El Salvador*" OR "Equatorial Guinea" OR Ecuador OR Fiji OR Gambia OR Guinea OR Ghana OR Gabon OR Georgia OR Grenada OR Guatemala OR Haiti OR Honduras OR India OR Indonesia OR Iran OR Iraq OR (West AND Bank) OR Gaza OR Jordan OR Jamaica OR "Democratic People's Republic of Korea" OR "North Korea" OR Kenya OR Kiribati OR Kyrgyzstan OR Kyrgyz OR Kazakhstan OR Kosovo OR Liberia OR "Lao PDR" OR Lebanon OR Lesotho OR Libya OR Madagascar OR Malawi OR Mali OR Mozambique OR Mauritania OR Micronesia OR Mongolia OR Morocco OR Myanmar OR Malaysia OR Maldives OR "Marshall Islands" OR Mauritius OR Mexico OR Moldova OR Montenegro OR Niger OR Nepal OR Nicaragua OR Nigeria OR Namibia OR "Republic of North Macedonia" OR Macedonia OR Pakistan OR "Papua New Guinea" OR Philippines OR Paraguay OR Peru OR Rwanda OR "Sierra Leone" OR Sierra OR Somalia OR "South Sudan" OR Sudan OR Syria OR Samoa OR "Sao Tome and Principe" OR Senegal OR Melanesia OR "Solomon Islands" OR "Sri Lanka" OR Serbia OR "South Africa" OR "South Africa*" OR "Saint Lucia" OR "Saint Vincent and the Grenadines" OR Suriname OR Togo OR Tanzania OR Tajikistan OR Timor-Leste OR Timor OR Tunisia OR Thailand OR Tonga OR Turkey OR Turkmen*)

AND (english[Filter])

File S2- Newcastle-Ottawa Quality Assessment scale

<u>Adapted Newcastle-Ottawa quality assessment scale for cross-sectional studies</u>

This scale has been adapted from the Newcastle-Ottawa scale created for cross-sectional studies by Herzog et al.

Note: A study can be awarded a maximum of one star for each numbered item within the Selection and Outcome categories. A maximum of two stars can be given for comparability.

Selection (Maximum 5 stars)

1. <u>Representativeness of the sample:</u>

a. Truly representative of the average in the target population (all subjects or random sampling) *

b. Somewhat representative of the average in the target group (non-random sampling) *

c. Selected group of users/convenience sample.

d. No description of the sampling strategy

2. <u>Sample size:</u>

a. Justified and satisfactory *

b. Not justified.

c. No information provided

3. <u>Non-respondents:</u>

a. comparability between respondents and non-respondents characteristics is established, and the response rate is satisfactory *

b. The response rate is unsatisfactory, or the comparability between respondents and non-respondents is unsatisfactory

c. No description of the response rate or the characteristics of the responders and the non-responders

4. Ascertainment of the exposure (risk factor):

a. Medical records or validated measurement tool **

b. Self-report **

c. No description of the measurement tool

Comparability (Maximum 2 stars)

Subjects in different study groups are comparable based on study design or analysis. Confounding factors are controlled.

1. Data/ results adjusted for most important factor - age *

2. Data/results adjusted for other important factors- education, wealth index, parity *

Outcome: (Maximum 3 stars)

1. Assessment of outcome:

a. Medical records/ by qualified medical personnel **

b. Self-report *

c. No description

2. Statistical test:

a. Statistical test used to analyse the data clearly described, appropriate and measures of association presented including confidence intervals and probability level (p-value) *

b. Statistical test not appropriate, not described, or incomplete.

Adapted Newcastle-Ottawa quality assessment scale for cohort studies

Note: A study can be awarded a maximum of one star for each numbered item within the Selection and Outcome categories. A maximum of two stars can be given for comparability

Selection (Maximum 4 stars)

1) Representativeness of the exposed cohort

a) truly representative of the average in the target population*

b) somewhat representative of the average in the target population *

c) selected group

d) no description of the derivation of the cohort

2) Selection of the non-exposed cohort

a) drawn from the same community as the exposed cohort *

b) drawn from a different source

c) no description of the derivation of the non-exposed cohort

3) Ascertainment of exposure

a) secure record (e.g., surgical records) *

b) structured interview *

c) written self-report

d) no description

4) Demonstration that outcome of interest was not present at start of study

a) yes *

b) no

Comparability (Maximum 2 stars)

1) Comparability of cohorts on the basis of the design or analysis

a) study controls for age*

b) study controls for additional factors- education, wealth index, parity*

Outcome

1) Assessment of outcome (Maximum 3 stars)

a) Independent blind assessment*

b) Record linkage*

b) self-report

c) no description

2) Was follow-up long enough for outcomes to occur

a) yes *

b) no

3) Adequacy of follow-up of cohorts

a) complete follow-up - all subjects accounted for *

b) Subjects lost to follow-up unlikely to introduce bias- number lost less than or equal to 20% or description of those lost suggested no different from those followed *

c) Follow-up rate less than 80% and no description of those lost

d) No statement

Table S1- Quality assessment of individual studies

Quality assessment- cross-sectional studies

Study	Selection				Comparability		Outcome		Total quality score
	Representativeness of the sample	Sample size	Non-respondents	Ascertainment of exposure	Adjusted for most important factor-age	Adjusted for other important factors- including education, wealth index, number of children	Assessment of outcome	Statistical test	
Escobar et al. (2016)	*	*	*	**	*	*	*	*	9
Liu et al. (2017)	*	*	*	**	*	*	**	*	10
Prusty et al. (2018)	*	*	*	**	*	*	*	*	9
Shekhar et al. (2019)	*	*	*	**	*	*	*		8
Desai et al. (2019)	*	*	*	**	*	*	*	*	9
P Geetha et al. (2019)	*	*		**	*		*	*	7
Meher and Sahoo (2019)	*	*	*	**	*	*	*		8
Ensor et al. (2020)	*	*	*	**	*	*	*		8
Singh et al. (2020)	*	*	*	**	*				6
Singh and Govil (2021)	*	*	*	**	*	*	*	*	9
Mozumdar (2021)	*	*	*	**	*	*	*	*	9
Kumari and Kundu (2022)	*	*	*	**	*	*	*	*	9
Desai et al. (2023)	*	*	*	**	*	*	*	*	9
Rout et al. (2023)	*	*	*	**	*	*	*	*	9
Singh et al. (2024)	*	*	*	**	*	*	*	*	9

Datta and Tiwari (2024)	*	*	*	**	*	*	*	*	9
Afonso et al. (2024)	*	*	*	**	*	*	*	*	9
Moosazadeh et al. (2024)	*	*	*	**	*	*	*	*	9
Singh V et al. (2024)	*	*	*	**	*	*	*	*	9

Quality assessment-cohort studies

Study	Selection				Comparability		Outcome			Total quality score
	Representativeness of the exposed cohort	Selection of the non-exposed cohort	Ascertainment of exposure	Demonstration that outcome not present at start of study	Controls for most important factor-age	Controls for additional factors-education, wealth index, parity	Assessment of outcome	Follow-up long enough for outcomes to occur	Adequacy of follow-up of cohorts	
Desai et al. (2017)	*	*	*	*	*	*		*	*	8
Jones et al. (2018)	*	*	*	*	*	*		*	*	8
Rajkumari et al. (2022)		*	*	*	*	*		*		6

Table S2- List of studies excluded after full-text review

Serial number	Study title	Author	Reason for exclusion
1	Rising premature menopause and variations by education level in India	Babbar et al. (2024)	Does not distinguish between natural menopause and surgical menopause
2	Epidemiological analysis of hysterectomies performed at the public health system in the largest Brazilian city	Augusto et al. (2021)	Describes surgical route, indications, cost, length of hospital stay, and not the factors associated with hysterectomy
3	The Socio-economic Status Predicting Women's Reproductive Health: A Prospective Cohort Study in Ardabil City, Iran, 2017-2020	Zandian et al. (2023)	Did not examine the association between socio-economic status and hysterectomy specifically. Classified women as having poor reproductive health based on based on history of hysterectomy, miscarriage, more than one abortion, or use of fertility medicines, and examined the association between socioeconomic status and poor reproductive health
4	The hysterectomy profile in Brazil (2009–2019): the epidemiology of a decade of public health data insights	Moretti-Marques et al. (2024)	Did not examine risk factors of hysterectomy
5	Prevalence of hysterectomy among rural and urban women with and without health insurance in Gujarat, India	Desai et al. (2011)	Did not report appropriate effect size measures

Table S3- Adjusted association between hysterectomy and sociodemographic factors in the included studies

Author (year)	Country	Design	Dataset	Variables adjusted for	Comparison group	Effect estimate	Effect estimate (95% CI)
	**AGE**						
Prusty (2018)	India	Cross-sectional	DLHS-4 (Married women aged 15-49 years in 21 states/UTs, non-Empowered Action Group states)	Children ever born, residence type, caste, religion, working status, insurance status, education, wealth index, tubal ligation	45-49 vs. <30	Odds ratio	8.60 (7.57-9.86)
Shekhar (2019)	India	Cross-sectional	NFHS-4 (Women aged 30-49 years)	Education, caste, religion, place of residence, wealth index, BMI, marital status, age at first cohabitation, parity, region	45-49 vs. 30-34	Odds ratio	3.84 (3.66-4.03)

Desai (2019)	India	Cross-sectional	NFHS-4 (Women aged 15-49 years)	Education, caste, religion, place of residence, wealth index, parity, tubal ligation	40-49 vs. 15-29	Odds ratio	16.9 (15.19-18.80)
Meher (2019)	India	Cross-sectional	NFHS-4 (Ever-married women aged 15-49 years, excluded gauna not performed)	Education, caste, religion, place of residence, wealth index, BMI, age at first cohabitation, parity, region, working status, tubal ligation	15-29 vs. 40-49	Odds ratio	0.09 (0.08-0.09)
Ensor (2020)	India (North-east)	Cross-sectional	NFHS-4 (Women aged 15-49 in the eight Northeastern states)	Education, parity, caste/tribe, marital status, insurance held, economic status; number of doctors, other medical staff, beds per 1000 population; distance between clusters of household and main surgical centres	40-45 vs. 15-19	Coefficient and t-statistic	0.529 (4.360)*
Singh A (2021)	India	Cross-sectional	NFHS-4 (Ever-married women aged 15-49 years)	Education, caste, religion, place of residence, wealth index, parity, tubal ligation, insurance, age at marriage	40-49 vs. 15-29	Odds ratio	14.71 (13.68-15.81)
Mozumdar (2021)	India	Cross-sectional	NFHS-4 (nonpregnant women aged between 30 and 49 years)	Education, social group, residence, wealth index, number of children, BMI, occupation status, region, age at first birth, anaemic status, ever use of contraception	Per 1 year increase	Odds ratio	1.09 (1.08-1.10)
Singh SK (2024)	India (Andhra Pradesh, Telangana, Bihar)	Cross-sectional	NFHS-4 & NFHS-5 (Women aged 15-49 years)	Years of schooling, caste/tribe, religion, place of residence, children ever born, wealth index, health insurance, ever use of family planning	40-49 vs. 15-29	Odds ratio	Andhra Pradesh: 10.17 (8.49, 12.18); Telangana: 22.81 (14.70, 35.40); Bihar: 10.36(8.65, 12.18)
Kumari (2022)	India	Cross-sectional	NFHS-5 (Women aged 15-49 years)	Education, caste, religion, place of residence, wealth index, parity, region, age at first cohabitation, marital status	40-49 vs. 15-29	Odds ratio	7.9 (7.33-8.65)

Desai (2023)	India	Cross-sectional	LASI (Women aged >=45 years)	Education, caste, religion, place of residence, monthly per capita expenditure (quintile), number of children, marital status, BMI category, ever employed for 3 or more months, state	60+ vs. 45-49	Odds ratio	0.92 (0.7, 1.19)
Rout (2023)	India	Cross-sectional	LASI (Women aged >18 years)	Age at marriage (in years), marital status, place of residence, caste, education, occupation, health insurance, MPCE quintile, number of children, physical activity, body mass index	≥60 vs. 18-44	Odds ratio	1.15 (0.82–1.63)
P Geetha (2019)	India (Andhra Pradesh)	Cross-sectional	Random sample of rural women in Chittoor (Women aged between 15 to 50 years)	- (only age adjusted)	>40 vs. 30-39	Prevalence odds ratio	7.741 (2.52,23.75)
Desai (2017)	India (Gujarat)	Cohort	A mixed-methods, population-based cohort study in Ahmedabad district (Women aged above 18 years)	Number of surviving children, income level, marital status, tubal ligation	55+ vs. 35-44	Rate ratio	0.03 (0.003-0.23)
Escobar (2016)	Latin America and Caribbean region	Cross-sectional	Health, Well-being and Aging Study (SABE)- Women aged 60 years and older	Marital status, education, household crowding, financial strain, health insurance, parity, ethnic background, cities	Per 1 year increase	Odds ratio	0.97 (0.96-0.98)
Afonso (2024)	Brazil	Cross-sectional	Brazilian Longitudinal Study of Ageing (ELSI- Brazil)- Women aged 50 years and above	Education, marital status, current smoker, preventive exam, number of births, hormone treatment, visited doctor in last 12 months, having a private health plan	Above 63 vs. 50-63	Prevalence ratio	1.02 (1.01-1.03)
Liu (2017)	China	Cross-sectional	Questionnaires collected from a subsample of an ongoing oesophageal cancer cohort study- residents aged 25-69 years in rural Anyang, China	BMI, parity, history of foetal loss	57-69 vs. 25-40	Odds ratio	10.10 (3.48-29.32)

Moosazadeh (2024)	Iran	Cross-sectional	TABARI cohort study (Women aged 35-70 years who reside in Sari, Mazandaran province in Northern Iran)	Education level, socioeconomic status, area of residence, has job, tubectomy, BMI, physical activity level, age at first pregnancy, number of pregnancies (gravida)	60-70 vs. 35-49	Odds ratio	5.83 (4.28–7.95)
	**EDUCATION**						
Prusty (2018)	India	Cross-sectional	DLHS-4 (Married women aged 15-49 years in 21 states/UTs, non-Empowered Action Group states)	Age at survey, children ever born, residence type, caste, religion, working status, insurance status, wealth index, tubal ligation	Matriculation or higher vs. No/ primary	Odds ratio	0.47(0.42-0.50)
Shekhar (2019)	India	Cross-sectional	NFHS-4 (Women aged 30-49 years)	Age, caste, religion, place of residence, wealth index, BMI, marital status, age at first cohabitation, parity, region	Higher vs. no schooling	Odds ratio	0.45 (0.41-0.49)
Desai (2019)	India	Cross-sectional	NFHS-4 (Women aged 15-49 years)	Age, caste, religion, place of residence, wealth index, parity, tubal ligation	10+ years of education vs. uneducated	Odds ratio	0.45 (0.40-0.49)
Meher (2019)	India	Cross-sectional	NFHS-4 (Ever-married women aged 15-49 years, excluded gauna not performed)	Age. caste, religion, place of residence, wealth index, BMI, age at first cohabitation, parity, region, working status, tubal ligation	Higher vs. no education	Odds ratio	0.40 (0.36-0.43)
Ensor (2020)	India (North-east)	Cross-sectional	NFHS-4 (Women aged 15-49 in the eight Northeastern states)	Age, parity, caste/tribe, marital status, insurance held, economic status; number of doctors, other medical staff, beds per 1000 population; distance between clusters of household and main surgical centres	Higher vs. no education	Coefficient and t-statistic	-0.253 (-0.980)
Singh A (2021)	India	Cross-sectional	NFHS-4 (Ever-married women aged 15-49 years)	Age, caste, religion, place of residence, wealth index, parity, sterilization, insurance, age at marriage	Higher vs. no education	Odds ratio	0.45 (0.41-0.49)

Mozumdar (2021)	India	Cross-sectional	NFHS-4 (nonpregnant women aged between 30 and 49 years)	Age (in years), social group, residence, wealth index, number of children, BMI, occupation status, region, age at first birth, anaemic status, ever use of contraception	No education vs. higher	Odds ratio	2.05 (1.63-2.59)
Singh SK (2024)	India (Andhra Pradesh, Telangana, Bihar)	Cross-sectional	NFHS-4 & NFHS-5 (Women aged 15-49 years)	Age, caste/tribe, religion, place of residence, children ever born, wealth index, health insurance, ever use of family planning	10 & above vs. no schooling	Odds ratio	Andhra Pradesh: 0.48(0.41,0.57); Telangana: 0.26(0.19,0.34); Bihar: 0.48(0.41,0.57)
Kumari (2022)	India	Cross-sectional	NFHS-5 (Women aged 15-49 years)	Age, caste, religion, place of residence, wealth index, parity, region, age at first cohabitation, marital status	Higher vs. no education	Odds ratio	0.4 (0.35-0.42)
Desai (2023)	India	Cross-sectional	LASI (Women aged >=45 years)	Age, caste, religion, place of residence, monthly per capita expenditure (quintile), number of children, marital status, BMI category, ever employed for 3 or more months, state	Middle school & higher vs. never attended school	Odds ratio	0.85 (0.56, 1.29)
Rout (2023)	India	Cross-sectional	LASI (Women aged >18 years)	Age, age at marriage (in years), marital status, place of residence, caste, occupation, health insurance, MPCE quintile, number of children, physical activity, body mass index	Graduate & above vs. no formal education	Odds ratio	1.36 (0.55–3.38)
P Geetha (2019)	India (Andhra Pradesh)	Cross-sectional	Random sample of rural women in Chittoor (Women aged between 15 to 50 years)	Age	Illiterate vs. secondary education	Prevalence odds ratio	3.82 (1.31,11.15)

Escobar (2016)	Latin America and Caribbean region	Cross-sectional	Health, Well-being and Aging Study (SABE)- Women aged 60 years and older	Age, marital status, household crowding, financial strain, health insurance, parity, ethnic background, cities	Years of education	Odds ratio	1.01(0.99-1.03)
Moosazadeh (2024)	Iran	Cross-sectional	TABARI cohort study (Women aged 35-70 years who reside in Sari, Mazandaran province in Northern Iran)	Age, socioeconomic status, area of residence, has job, tubectomy, BMI, physical activity level, age at first pregnancy, number of pregnancies (gravida)	No schooling vs. university/college	Odds ratio	1.30 (0.82–2.06)
	**PLACE OF RESIDENCE**						
Prusty (2018)	India	Cross-sectional	DLHS-4 (Married women aged 15-49 years in 21 states/UTs, non-Empowered Action Group states)	Age at survey, children ever born, caste, religion, working status, insurance status, education, wealth index, tubal ligation	Urban vs. rural	Odds ratio	0.99 (0.93-1.06)
Shekhar (2019)	India	Cross-sectional	NFHS-4 (Women aged 30-49 years)	Age, education, caste, religion, wealth index, BMI, marital status, age at first cohabitation, parity, region	Rural vs. urban	Odds ratio	1.46 (1.41-1.52)
Desai (2019)	India	Cross-sectional	NFHS-4 (Women aged 15-49 years)	Age, education, caste, religion, wealth index, parity, tubal ligation	Rural vs. urban	Odds ratio	1.36 (1.27-1.45)
Meher (2019)	India	Cross-sectional	NFHS-4 (Ever-married women aged 15-49 years, excluded gauna not performed)	Age. education, caste, religion, wealth index, BMI, age at first cohabitation, parity, region, working status, tubal ligation	Rural vs. urban	Odds ratio	1.29 (1.24-1.35)
Singh A (2021)	India	Cross-sectional	NFHS-4 (Ever-married women aged 15-49 years)	Age, education, caste, religion, wealth index, parity, sterilization, insurance, age at marriage	Rural vs. urban	Odds ratio	1.22 (1.16-1.28)
Mozumdar (2021)	India	Cross-sectional	NFHS-4 (nonpregnant women aged between 30 and 49 years)	Age (in years), education, social group, wealth index, number of children, BMI, occupation status, region, age at first birth, anaemic status, ever use of contraception	Rural vs. urban	Odds ratio	1.26 (1.15-1.39)

Singh SK (2024)	India (Andhra Pradesh, Telangana, Bihar)	Cross-sectional	NFHS-4 & NFHS-5 (Women aged 15-49 years)	Age, years of schooling, caste/tribe, religion, children ever born, wealth index, health insurance, ever use of family planning	Rural vs. urban	Odds ratio	Andhra Pradesh: 1.35(1.12,1.61); Telangana: 1.76 (1.45,2.13); Bihar: 1.35(1.12,1.61)
Kumari (2022)	India	Cross-sectional	NFHS-5 (Women aged 15-49 years)	Age, education, caste, religion, wealth index, parity, region, age at first cohabitation, marital status	Rural vs. urban	Odds ratio	1.3 (1.23-1.35)
Desai (2023)	India	Cross-sectional	LASI (Women aged >=45 years)	Age, education, caste, religion, monthly per capita expenditure (quintile), number of children, marital status, BMI category, ever employed for 3 or more months, state	Urban vs. rural	Odds ratio	1.32 (1.1, 1.59)
Rout (2023)	India	Cross-sectional	LASI (Women aged >18 years)	Age, age at marriage (in years), marital status, caste, education, occupation, health insurance, MPCE quintile, number of children, physical activity, body mass index	Urban vs. rural	Odds ratio	1.51 (1.21–1.88)
Moosazadeh (2024)	Iran	Cross-sectional	TABARI cohort study (Women aged 35-70 years who reside in Sari, Mazandaran province in Northern Iran)	Age, education level, socioeconomic status, has job, tubectomy, BMI, physical activity level, age at first pregnancy, number of pregnancies (gravida)	Mouintaneous vs. urban	Odds ratio	0.57 (0.43–0.75)
	**HOUSEHOLD INCOME**						
Prusty (2018)	India	Cross-sectional	DLHS-4 (Married women aged 15-49 years in 21 states/UTs, non-Empowered Action Group states)	Age at survey, children ever born, residence type, caste, religion, working status, insurance status, education, tubal ligation	Rich vs. poor	Odds ratio	1.48 (1.36-1.60)

Shekhar (2019)	India	Cross-sectional	NFHS-4 (Women aged 30-49 years)	Age, education, caste, religion, place of residence, BMI, marital status, age at first cohabitation, parity, region	Highest vs. lowest	Odds ratio	2.03 (1.90-2.17)
Desai (2019)	India	Cross-sectional	NFHS-4 (Women aged 15-49 years)	Age, education, caste, religion, place of residence, parity, tubal ligation	Rich vs. poor	Odds ratio	1.76 (1.65-1.88)
Meher (2019)	India	Cross-sectional	NFHS-4 (Ever-married women aged 15-49 years, excluded gauna not performed)	Age. education, caste, religion, place of residence, BMI, age at first cohabitation, parity, region, working status, tubal ligation	Richest vs. poorest	Odds ratio	2.36*
Ensor (2020)	India (North-east)	Cross-sectional	NFHS-4 (Women aged 15-49 in the eight Northeastern states)	Age, education, parity, caste/tribe, marital status, insurance held, number of doctors, other medical staff, beds per 1000 population; distance between clusters of household and main surgical centers	Richest vs. poorest	Coefficient and t-statistic	1.330 (5.480)*
Singh A (2021)	India	Cross-sectional	NFHS-4 (Ever-married women aged 15-49 years)	Age, education, caste, religion, place of residence, parity, sterilization, insurance, age at marriage	Richest vs. poorest	Odds ratio	2.67 (2.47-2.88)
Mozumdar (2021)	India	Cross-sectional	NFHS-4 (nonpregnant women aged between 30 and 49 years)	Age (in years), education, social group, residence, number of children, BMI, occupation status, region, age at first birth, anaemic status, ever use of contraception	Richest vs. middle	Odds ratio	1.23 (1.08-1.41)

Singh SK (2024)	India (Andhra Pradesh, Telangana, Bihar)	Cross-sectional	NFHS-4 & NFHS-5 (Women aged 15-49 years)	Age, years of schooling, caste/tribe, religion, place of residence, children ever born, health insurance, ever use of family planning	Richest vs. poorest	Odds ratio	Andhra Pradesh: 2.18(1.81,2.63); Telangana: 2.57(1.94,3.42); Bihar: 2.18(1.81,2.63)
Kumari (2022)	India	Cross-sectional	NFHS-5 (Women aged 15-49 years)	Age, education, caste, religion, place of residence, parity, region, age at first cohabitation, marital status	Richest vs. poorest	Odds ratio	2.6 (2.37-2.76)
Desai (2023)	India	Cross-sectional	LASI (Women aged >=45 years)	Age, education, caste, religion, place of residence, number of children, marital status, BMI category, ever employed for 3 or more months, state	Highest wealth quintile vs. lowest	Odds ratio	1.76 (1.32, 2.34)
Rout (2023)	India	Cross-sectional	LASI (Women aged >18 years)	Age, age at marriage (in years), marital status, place of residence, caste, education, occupation, health insurance, number of children, physical activity, body mass index	Rich vs. poor	Odds ratio	1.82 (1.51–2.19)
P Geetha (2019)	India (Andhra Pradesh)	Cross-sectional	Random sample of rural women in Chittoor (Women aged between 15 to 50 years)	Age	Low (<Rs 49999) vs. Middle (Rs 50000-99999)	Prevalence odds ratio	3.29 (0.95,11.41)
Desai (2017)	India (Gujarat)	Cohort	A mixed-methods, population-based cohort study in Ahmedabad district	Number of surviving children, age at start of follow-up, marital status, tubal ligation	Rs. 120001+ vs. Rs. 0-60,000	Rate ratio	0.12 (0.03-0.45)
Escobar (2016)	Latin America and Caribbean region	Cross-sectional	Health, Well-being and Aging Study (SABE)- Women aged 60 years and older	Age, marital status, education, household crowding, health insurance, parity, ethnic background, cities	Financial strain: yes vs. no	Odds ratio	0.92 (0.79-1.06)
Moosazadeh (2024)	Iran	Cross-sectional	TABARI cohort study (Women aged 35-70 years who reside in	Age, education level, area of residence, has job, tubectomy,	Socioeconomic level: 5 vs. 1	Odds ratio	1.66 (1.13–2.42)

			Sari, Mazandaran province in Northern Iran)	BMI, physical activity level, age at first pregnancy, number of pregnancies (gravida)			
	**EMPLOYMENT STATUS**						
Prusty (2018)	India	Cross-sectional	DLHS-4 (Married women aged 15-49 years in 21 states/UTs, non-Empowered Action Group states)	Age at survey, children ever born, residence type, caste, religion, insurance status, education, wealth index, tubal ligation	Working vs. not working	Odds ratio	1.47 (1.38-1.57)
Meher (2019)	India	Cross-sectional	NFHS-4 (Ever-married women aged 15-49 years, excluded gauna not performed)	Age. education, caste, religion, place of residence, wealth index, BMI, age at first cohabitation, parity, region, tubal ligation	Working vs. not working	Odds ratio	1.053
Mozumdar (2021)	India	Cross-sectional	NFHS-4 (nonpregnant women aged between 30 and 49 years)	Age (in years), education, social group, residence, wealth index, number of children, BMI, region, age at first birth, anaemic status, ever use of contraception	Farming vs. not working	Odds ratio	1.26 (1.13-1.39)
Desai (2023)	India	Cross-sectional	LASI (Women aged >=45 years)	Age, education, caste, religion, place of residence, monthly per capita expenditure (quintile), number of children, marital status, BMI category, state	Ever employed for 3 or more months: yes vs. no	Odds ratio	1.09 (0.9, 1.32)
Rout (2023)	India	Cross-sectional	LASI (Women aged >18 years)	Age, age at marriage (in years), marital status, place of residence, caste, education, health insurance, MPCE quintile, number of children, physical activity, body mass index	Working vs. not working	Odds ratio	1.17 (1.01–1.37)

Moosazadeh (2024)	Iran	Cross-sectional	TABARI cohort study (Women aged 35-70 years who reside in Sari, Mazandaran province in Northern Iran)	Age, education level, socioeconomic status, area of residence, tubectomy, BMI, physical activity level, age at first pregnancy, number of pregnancies (gravida)	Working vs. not working	Odds ratio	0.62 (0.45–0.86)
	**MARITAL STATUS**						
Shekhar (2019)	India	Cross-sectional	NFHS-4 (Women aged 30-49 years)	Age, education, caste, religion, place of residence, wealth index, BMI, age at first cohabitation, parity, region	Others (excluding widowed) vs. currently married	Odds ratio	1.20 (0.58-2.45)
Ensor (2020)	India (North-east)	Cross-sectional	NFHS-4 (Women aged 15-49 in the eight Northeastern states)	Age, education, parity, caste/tribe, insurance held, economic status; number of doctors, other medical staff, beds per 1000 population; distance between clusters of household and main surgical centres	Married vs. never married	Coefficient and t-statistic	1.074 (2.400) *
Kumari (2022)	India	Cross-sectional	NFHS-5 (Women aged 15-49 years)	Age, education, caste, religion, place of residence, wealth index, parity, region, age at first cohabitation	Others (excluding widowed) vs. currently married	Odds ratio	0.2 (0.19-0.24)
Desai (2023)	India	Cross-sectional	LASI (Women aged >=45 years)	Age, education, caste, religion, place of residence, monthly per capita expenditure (quintile), number of children, BMI category, ever employed for 3 or more months, state	Never married vs. currently married	Odds ratio	0.15 (0.04, 0.59)
Desai (2017)	India (Gujarat)	Cohort	A mixed-methods, population-based cohort study in Ahmedabad district	Number of surviving children, income level, age at start of follow-up, tubal ligation	Unmarried vs. married	Rate ratio	0.18(0.02-1.90)

Escobar (2016)	Latin America and Caribbean region	Cross-sectional	Health, Well-being and Aging Study (SABE)- Women aged 60 years and older	Age, education, household crowding, financial strain, health insurance, parity, ethnic background, cities	Married vs. unmarried	Odds ratio	1.10 (0.96-1.28)
Afonso (2024)	Brazil	Cross-sectional	Brazilian Longitudinal Study of Ageing (ELSI- Brazil)- Women aged 50 years and above	Age, education, current smoker, preventive exam, number of births, hormone treatment, visited doctor in last 12 months, having a private health plan	With partner vs. no partner	Prevalence ratio	1.02 (1.01-1.03)
	**BODY MASS INDEX (BMI)**						
Shekhar (2019)	India	Cross-sectional	NFHS-4 (Women aged 30-49 years)	Age, education, caste, religion, place of residence, wealth index, marital status, age at first cohabitation, parity, region	>=25 kg/m^2 vs. <25 kg/m^2	Odds ratio	1.39 (1.35-1.44)
Meher (2019)	India	Cross-sectional	NFHS-4 (Ever-married women aged 15-49 years, excluded gauna not performed)	Age. education, caste, religion, place of residence, wealth index, age at first cohabitation, parity, region, working status, tubal ligation	Obese (>=25 kg/m^2) vs. thin (<18.5 kg/m^2)	Odds ratio	1.43 (1.35-1.51)
Mozumdar (2021)	India	Cross-sectional	NFHS-4 (nonpregnant women aged between 30 and 49 years)	Age (in years), education, social group, residence, wealth index, number of children, occupation status, region, age at first birth, anaemic status, ever use of contraception	Obese vs. normal	Odds ratio	1.44 (1.26- 1.65)
Desai (2023)	India	Cross-sectional	LASI (Women aged >=45 years)	Age, education, caste, religion, place of residence, monthly per capita expenditure (quintile), number of children, marital status, ever employed for 3 or more months, state	Obese vs. normal	Odds ratio	1.1 (0.81, 1.5)
Rout (2023)	India	Cross-sectional	LASI (Women aged >18 years)	Age, age at marriage (in years), marital status, place of	Obese vs. underweight	Odds ratio	2.43 (1.77–3.32)

				residence, caste, education, occupation, health insurance, MPCE quintile, number of children, physical activity			
Rajkumari (2022)	India (Haryana)	Cohort	A cohort study among ever-married women aged 35-70 years from the Jat community from Palwal district	Age, educational status, occupation, smoking, alcoholism, age at menarche, age at first conception, age at last conception, history of foetal loss, tubal ligation, Hip circumference, Blood pressure, lipid parameters	Obese vs. normal	Odds ratio	1.05 (0.54-2.03)
Liu (2017)	China	Cross-sectional	Questionnaires collected from a subsample of an ongoing oesophagal cancer cohort study-residents aged 25-69 years in rural Anyang, China	Age, parity, history of foetal loss	Obese (>=28 kg/m^2) vs. normal (18.5-<24 kg/m^2)	Odds ratio	1.59(0.99-2.56)
Moosazadeh (2024)	Iran	Cross-sectional	TABARI cohort study (Women aged 35-70 years who reside in Sari, Mazandaran province in Northern Iran)	Age, education level, socioeconomic status, area of residence, has job, tubectomy, physical activity level, age at first pregnancy, number of pregnancies (gravida)	>=30 kg/m^2 vs. <25 kg/m^2	Odds ratio	0.92 (0.69–1.22)
	**HEALTH INSURANCE**						
Prusty (2018)	India	Cross-sectional	DLHS-4 (Married women aged 15-49 years in 21 states/UTs, non-Empowered Action Group states)	Age at survey, children ever born, residence type, caste, religion, working status, education, wealth index, tubal ligation	Yes vs. no	Odds ratio	1.88(1.77-2.00)
Ensor (2020)	India (North-east)	Cross-sectional	NFHS-4 (Women aged 15-49 in the eight Northeastern states)	Age, education, parity, caste/tribe, marital status, economic status, number of doctors, other medical staff,	State, RSBY, Other insurance vs. no insurance	Coefficient and t-statistic	State- 0.017 (0.120); RSBY- 0.092 (0.470); Other- 0.008 (0.020)

				beds per 1000 population; distance between clusters of household and main surgical centres			
Singh A (2021)	India	Cross-sectional	NFHS-4 (Ever-married women aged 15-49 years)	Age, education, caste, religion, place of residence, wealth index, parity, sterilization, age at marriage	Yes vs. no	Odds ratio	1.16 (1.11-1.22)
Singh V (2024)	India (Andhra Pradesh)	Cross-sectional	NFHS-4	Propensity score estimation- age, education, obstetric history, undergone sterilisation, parity, wealth index, social category, religion, rural/urban residence, presence of non-communicable diseases	Coverage in Andhra Pradesh's state-specific publicly funded health insurance (Aarogyasri scheme) vs. otherwise	Average Treatment effect on the Treated (ATT)	Nearest neighbour 1:1- 0.107 (0.029)*
Singh SK (2024)	India (Andhra Pradesh, Telangana, Bihar)	Cross-sectional	NFHS-4 & NFHS-5 (Women aged 15-49 years)	Age, years of schooling, caste/tribe, religion, place of residence, children ever born, wealth index, ever use of family planning	Yes vs. no	Odds ratio	Andhra Pradesh: 1.21(1.07,1.38); Telangana: 1.05 (0.93,1.20); Bihar: 1.21(1.07,1.38)
Rout (2023)	India	Cross-sectional	LASI (Women aged >18 years)	Age, age at marriage (in years), marital status, place of residence, caste, education, occupation, MPCE quintile, number of children, physical activity, body mass index	Yes vs. no	Odds ratio	1.14 (0.94–1.38)
Afonso (2024)	Brazil	Cross-sectional	Brazilian Longitudinal Study of Ageing (ELSI- Brazil)- Women aged 50 years and above	Age, education, marital status, current smoker, preventive exam, number of births, hormone treatment, visited doctor in last 12 months	Private health plan: yes vs. no	Prevalence ratio	1.02 (1.01-1.03)

DLHS- District Level Household and Facility Survey; LASI- Longitudinal Ageing Study in India; NFHS- National Family Health Survey

Table S4- Adjusted association between hysterectomy and reproductive factors in the included studies

Author (year)	Country	Design	Dataset	Variables adjusted for	Comparison group	Effect estimate	Effect estimate (95% CI)
				**NUMBER OF CHILDREN**			
Prusty (2018)	India	Cross-sectional	DLHS-4 (Married women aged 15-49 years in 21 states/UTs, non-Empowered Action Group states)	Age at survey, residence type, caste, religion, working status, insurance status, education, wealth index, tubal ligation	2+ vs. 0	Odds ratio	1.74 (1.41-2.14)
Shekhar (2019)	India	Cross-sectional	NFHS-4 (Women aged 30-49 years)	Age, education, caste, religion, place of residence, wealth index, BMI, marital status, age at first cohabitation, region	3 and above vs. 0	Odds ratio	1.85 (1.66-2.06)
Desai (2019)	India	Cross-sectional	NFHS-4 (Women aged 15-49 years)	Age, education, caste, religion, place of residence, wealth index, tubal ligation	3+ vs. 0-1	Odds ratio	2.43 (2.21-2.68)
Meher (2019)	India	Cross-sectional	NFHS-4 (Ever-married women aged 15-49 years, excluded gauna not performed)	Age. education, caste, religion, place of residence, wealth index, BMI, age at first cohabitation, region, working status, tubal ligation	3 and above vs. 0	Odds ratio	2.839*
Ensor (2020)	India (North-east)	Cross-sectional	NFHS-4 (Women aged 15-49 in the eight Northeastern states)	Age, education, caste/tribe, marital status, insurance held, economic status, number of doctors, other medical staff, beds per 1000 population, distance between clusters of household and main surgical centres	4 or more vs. one	Coefficient and t-statistic	-0.145 (-0.780)
Singh A (2021)	India	Cross-sectional	NFHS-4 (Ever-married women aged 15-49 years)	Age, education, caste, religion, place of residence, wealth index, tubal ligation, insurance, age at marriage	3 or more vs. 0	Odds ratio	2.74 (2.42-3.11)

Mozumdar (2021)	India	Cross-sectional	NFHS-4 (nonpregnant women aged between 30 and 49 years)	Age (in years), education, social group, residence, wealth index, BMI, occupation status, region, age at first birth, anaemic status, ever use of contraception	4+ vs. 0	Odds ratio	1.32 (0.99-1.77)
Singh SK (2024)	India (Andhra Pradesh, Telangana, Bihar)	Cross-sectional	NFHS-4 & NFHS-5 (Women aged 15-49 years)	Age, years of schooling, caste/tribe, religion, place of residence, wealth index, health insurance, ever use of family planning	4+ vs. 1	Odds ratio	Andhra Pradesh: 3.69(2.70,5.03), Telangana: 1.84(1.42,2.39), Bihar: 3.69(2.70,5.03)
Kumari (2022)	India	Cross-sectional	NFHS-5 (Women aged 15-49 years)	Age, education, caste, religion, place of residence, wealth index, region, age at first cohabitation, marital status	3 and above vs. 0	Odds ratio	2.9 (2.55-3.18)
Desai (2023)	India	Cross-sectional	LASI (Women aged >=45 years)	Age, education, caste, religion, place of residence, monthly per capita expenditure (quintile), marital status, BMI category, ever employed for 3 or more months, state	3 or more vs. 0	Odds ratio	1.25 (0.83, 1.88)
Rout (2023)	India	Cross-sectional	LASI (Women aged >18 years)	Age, age at marriage (in years), marital status, place of residence, caste, education, occupation, health insurance, MPCE quintile, physical activity, body mass index	Multiparous vs. uniparous	Odds ratio	1.58 (0.95–2.62)
P Geetha (2019)	India (Andhra Pradesh)	Cross-sectional	Random sample of rural women in Chittoor (Women aged between 15 to 50 years)	Age	Total conceptions: >4 vs. 1	Prevalence odds ratio	2.09 (0.40,11.00)
Desai (2017)	India (Gujarat)	Cohort	A mixed-methods, population-based cohort study in Ahmedabad district	Income level, age at start of follow-up, marital status, tubal ligation	4+ vs. 2-3	Rate ratio	0.81 (0.40,1.66)

Escobar (2016)	Latin America and Caribbean region	Cross-sectional	Health, Well-being and Aging Study (SABE)- Women aged 60 years and older	Age, marital status, education, household crowding, financial strain, health insurance, ethnic background, cities	>=3 vs. 0-2	Odds ratio	0.79 (0.67-0.92)
Afonso (2024)	Brazil	Cross-sectional	Brazilian Longitudinal Study of Ageing (ELSI- Brazil)- Women aged 50 years and above	Age, education, marital status, current smoker, preventive exam, hormone treatment, visited doctor in last 12 months, having a private health plan	Up to 3 vs. more than 3	Prevalence ratio	1.02 (1.01-1.03)
Liu (2017)	China	Cross-sectional	Questionnaires collected from a subsample of an ongoing oesophagal cancer cohort study- residents aged 25-69 years in rural Anyang, China	Age, BMI, history of foetal loss	>2 vs. <=2	Odds ratio	1.05(0.67-1.64)
Moosazadeh (2024)	Iran	Cross-sectional	TABARI cohort study (Women aged 35-70 years who reside in Sari, Mazandaran province in Northern Iran)	Age, education level, socioeconomic status, area of residence, has job, tubectomy, BMI, physical activity level, age at first pregnancy	Number of pregnancies: >=5 vs. 0-1	Odds ratio	5.35 (1.62–17.63)
			**TUBAL LIGATION**				
Prusty (2018)	India	Cross-sectional	DLHS-4 (Married women aged 15-49 years in 21 states/UTs, non-Empowered Action Group states)	Age at survey, children ever born, residence type, caste, religion, working status, insurance status, education, wealth index	Yes vs. no	Odds ratio	1.55(1.45-1.67)
Desai (2019)	India	Cross-sectional	NFHS-4 (Women aged 15-49 years)	Age, education, caste, religion, place of residence, wealth index, parity	Yes vs. no	Odds ratio	0.64 (0.61-0.68)
Meher (2019)	India	Cross-sectional	NFHS-4 (Ever-married women aged 15-49 years, excluded gauna not performed)	Age. education, caste, religion, place of residence, wealth index, BMI, age at first cohabitation, parity, region, working status	Yes vs. no	Odds ratio	0.628*

Singh A (2021)	India	Cross-sectional	NFHS-4 (Ever-married women aged 15-49 years)	Age, education, caste, religion, place of residence, wealth index, parity, insurance, age at marriage	Yes vs. no	Odds ratio	0.65 (0.63-0.68)
Desai (2017)	India (Gujarat)	Cohort	A mixed-methods, population-based cohort study in Ahmedabad district	Number of surviving children, income level, age at start of follow-up, marital status	No vs. yes	Rate ratio	0.54 (0.19-1.54)
Rajkumari (2022)	India (Haryana)	Cohort	A cohort study among ever-married women aged 35-70 years from the Jat community from Palwal district	Age, educational status, occupation, smoking, alcoholism, age at menarche, age at first conception, age at last conception, history of foetal loss, BMI, Hip circumference, Blood pressure, lipid parameters	Yes vs. no	Odds ratio	1.24 (0.69-2.22)
Moosazadeh (2024)	Iran	Cross-sectional	TABARI cohort study (Women aged 35-70 years who reside in Sari, Mazandaran province in Northern Iran)	Age, education level, socioeconomic status, area of residence, has job, BMI, physical activity level, age at first pregnancy, number of pregnancies (gravida)	Yes vs. no	Odds ratio	1.27 (1.05–1.53)
	**AGE AT FIRST BIRTH**						
Mozumdar (2021)	India	Cross-sectional	NFHS-4 (nonpregnant women aged between 30 and 49 years)	Age (in years), education, social group, residence, wealth index, number of children, BMI, occupation status, region, anaemic status, ever use of contraception	<16 years vs. >=25 years	Odds ratio	3.07 (2.56-3.68)
Datta (2024)	India	Cross-sectional	NFHS-5 (Ever-married women aged 20-49 years)	Child marriage, adolescent childbirth, age, place of residence, wealth index, education, religion, caste, BMI, parity, state fixed effects	<=19 years vs. >19 years	Odds ratio	1.53 (1.40-1.66)
P Geetha (2019)	India (Andhra Pradesh)	Cross-sectional	Random sample of rural women in Chittoor (Women aged between 15 to 50 years)	Age	Age at first conception: <18	Odds ratio	1.60 (0.49,5.22)

					years vs. 22-24 years		
Rajkumari (2022)	India (Haryana)	Cohort	A cohort study among ever-married women aged 35-70 years from the Jat community from Palwal district	Age, educational status, occupation, smoking, alcoholism, age at menarche, age at last conception, history of foetal loss, tubal ligation, BMI, Hip circumference, Blood pressure, lipid parameters	<=18 years vs. 19-30 years	Odds ratio	1.15 (0.67–1.97)
Moosazadeh (2024)	Iran	Cross-sectional	TABARI cohort study (Women aged 35-70 years who reside in Sari, Mazandaran province in Northern Iran)	Age, education level, socioeconomic status, area of residence, has job, tubectomy, BMI, physical activity level, number of pregnancies (gravida)	Age at first pregnancy: <20 years vs. without pregnancy	Odds ratio	0.76 (0.19-3.00)

DLHS- District Level Household and Facility Survey; LASI- Longitudinal Ageing Study in India; NFHS- National Family Health Survey

The PRISMA checklist

Section and Topic	Item #	Checklist item	Location where item is reported
**TITLE**			
Title	1	Identify the report as a systematic review.	Page 1- Title
**ABSTRACT**			
Abstract	2	See the PRISMA 2020 for Abstracts checklist.	Page 2
**INTRODUCTION**			
Rationale	3	Describe the rationale for the review in the context of existing knowledge.	Page 3-4
Objectives	4	Provide an explicit statement of the objective(s) or question(s) the review addresses.	Page 4
**METHODS**			
Eligibility criteria	5	Specify the inclusion and exclusion criteria for the review and how studies were grouped for the syntheses.	Page 4-5
Information sources	6	Specify all databases, registers, websites, organisations, reference lists and other sources searched or consulted to identify studies. Specify the date when each source was last searched or consulted.	Page 4
Search strategy	7	Present the full search strategies for all databases, registers and websites, including any filters and limits used.	File S1 in Online Supplementary Document
Selection process	8	Specify the methods used to decide whether a study met the inclusion criteria of the review, including how many reviewers screened each record and each report retrieved, whether they worked independently, and if applicable, details of automation tools used in the process.	Page 5
Data collection process	9	Specify the methods used to collect data from reports, including how many reviewers collected data from each report, whether they worked independently, any processes for obtaining or confirming data from study investigators, and if applicable, details of automation tools used in the process.	Page 5
Data items	10a	List and define all outcomes for which data were sought. Specify whether all results that were compatible with each outcome domain in each study were sought (e.g. for all measures, time points, analyses), and if not, the methods used to decide which results to collect.	Page 5
	10b	List and define all other variables for which data were sought (e.g. participant and intervention characteristics, funding sources).	Page 5-6

Section and Topic	Item #	Checklist item	Location where item is reported
		Describe any assumptions made about any missing or unclear information.	
Study risk of bias assessment	11	Specify the methods used to assess risk of bias in the included studies, including details of the tool(s) used, how many reviewers assessed each study and whether they worked independently, and if applicable, details of automation tools used in the process.	Page 5, File S2 in Online Supplementary Document
Effect measures	12	Specify for each outcome the effect measure(s) (e.g. risk ratio, mean difference) used in the synthesis or presentation of results.	Page 5-6
Synthesis methods	13a	Describe the processes used to decide which studies were eligible for each synthesis (e.g. tabulating the study intervention characteristics and comparing against the planned groups for each synthesis (item #5)).	Table 1, page 5
	13b	Describe any methods required to prepare the data for presentation or synthesis, such as handling of missing summary statistics, or data conversions.	Page 5-6
	13c	Describe any methods used to tabulate or visually display results of individual studies and syntheses.	Page 5, Table 1, Table S3 and Table S4 in Online Supplementary Document
	13d	Describe any methods used to synthesize results and provide a rationale for the choice(s). If meta-analysis was performed, describe the model(s), method(s) to identify the presence and extent of statistical heterogeneity, and software package(s) used.	Page 5
	13e	Describe any methods used to explore possible causes of heterogeneity among study results (e.g. subgroup analysis, meta-regression).	Page 5-6
	13f	Describe any sensitivity analyses conducted to assess robustness of the synthesized results.	Page 5-6
Reporting bias assessment	14	Describe any methods used to assess risk of bias due to missing results in a synthesis (arising from reporting biases).	-
Certainty assessment	15	Describe any methods used to assess certainty (or confidence) in the body of evidence for an outcome.	Page 5-6
**RESULTS**			
Study selection	16a	Describe the results of the search and selection process, from the number of records identified in the search to the number of studies included in the review, ideally using a flow diagram.	Page 6, Figure 1
	16b	Cite studies that might appear to meet the inclusion criteria, but which were excluded, and explain why they were excluded.	Table S2 in Online Supplementary Document
Study characteristics	17	Cite each included study and present its characteristics.	Page 6, Table 1

Section and Topic	Item #	Checklist item	Location where item is reported
Risk of bias in studies	18	Present assessments of risk of bias for each included study.	Page 6, Table S1 in Online Supplementary Document
Results of individual studies	19	For all outcomes, present, for each study: (a) summary statistics for each group (where appropriate) and (b) an effect estimate and its precision (e.g. confidence/credible interval), ideally using structured tables or plots.	Table S3 and Table S4 in Online Supplementary Document
Results of syntheses	20a	For each synthesis, briefly summarise the characteristics and risk of bias among contributing studies.	Page 7-11
	20b	Present results of all statistical syntheses conducted. If meta-analysis was done, present for each the summary estimate and its precision (eg. confidence/credible interval) and measures of statistical heterogeneity. If comparing groups, describe the direction of the effect.	Page 7-11
	20c	Present results of all investigations of possible causes of heterogeneity among study results.	Page 7
	20d	Present results of all sensitivity analyses conducted to assess the robustness of the synthesized results.	Page 7-11
Reporting biases	21	Present assessments of risk of bias due to missing results (arising from reporting biases) for each synthesis assessed.	-
Certainty of evidence	22	Present assessments of certainty (or confidence) in the body of evidence for each outcome assessed.	Page 7-11
**DISCUSSION**			
Discussion	23a	Provide a general interpretation of the results in the context of other evidence.	Page 11-15
	23b	Discuss any limitations of the evidence included in the review.	Page 15-16
	23c	Discuss any limitations of the review processes used.	Page 15-16
	23d	Discuss implications of the results for practice, policy, and future research.	Page 14-15
**OTHER INFORMATION**			
Registration and protocol	24a	Provide registration information for the review, including register name and registration number, or state that the review was not registered.	Page 4
	24b	Indicate where the review protocol can be accessed, or state that a protocol was not prepared.	Page 4
	24c	Describe and explain any amendments to information provided at registration or in the protocol.	-
Support	25	Describe sources of financial or non-financial support for the review, and the role of the funders or sponsors in the review.	Page 17
Competing	26	Declare any competing interests of review authors.	Page 17

Section and Topic	Item #	Checklist item	Location where item is reported
interests			
Availability of data, code and other materials	27	Report which of the following are publicly available and where they can be found: template data collection forms; data extracted from included studies; data used for all analyses; analytic code; any other materials used in the review.	Online Supplementary Document

From: Page MJ, McKenzie JE, Bossuyt PM, Boutron I, Hoffmann TC, Mulrow CD, et al. The PRISMA 2020 statement: an updated guideline for reporting systematic reviews. BMJ 2021;372:n71. doi: 10.1136/bmj.n71

The Synthesis Without Meta-analysis (SWiM) checklist

**SWiM is intended to complement and be used as an extension to PRISMA**			
**SWiM reporting item**	**Item description**	**Page in manuscript where item is reported**	**Other***
Methods			
**1** Grouping studies for synthesis	1a) Provide a description of, and rationale for, the groups used in the synthesis (e.g., groupings of populations, interventions, outcomes, study design)	Page 5-6	
	1b) Detail and provide rationale for any changes made subsequent to the protocol in the groups used in the synthesis	-	
**2** Describe the standardised metric and transformation methods used	Describe the standardised metric for each outcome. Explain why the metric(s) was chosen, and describe any methods used to transform the intervention effects, as reported in the study, to the standardised metric, citing any methodological guidance consulted	Page 5. Studies that reported quantitative information was included. Since a narrative synthesis of the findings were undertaken, no specific standardized metric was chosen.	
**3** Describe the synthesis methods	Describe and justify the methods used to synthesise the effects for each outcome when it was not possible to undertake a meta-analysis of effect estimates	Page 5-6	
**4** Criteria used to prioritise results for summary and synthesis	Where applicable, provide the criteria used, with supporting justification, to select the particular studies, or a particular study, for the main synthesis or to draw conclusions from the synthesis (e.g., based on study design, risk of bias assessments, directness in relation to the review question)	Page 5-6	
**SWiM reporting item**	**Item description**	**Page in manuscript where item is reported**	**Other***

**5** Investigation of heterogeneity in reported effects	State the method(s) used to examine heterogeneity in reported effects when it was not possible to undertake a meta-analysis of effect estimates and its extensions to investigate heterogeneity	Pages 5,6	
**6** Certainty of evidence	Describe the methods used to assess certainty of the synthesis findings	Pages 5,6	
**7** Data presentation methods	Describe the graphical and tabular methods used to present the effects (e.g., tables, forest plots, harvest plots).<br><br>Specify key study characteristics (e.g., study design, risk of bias) used to order the studies, in the text and any tables or graphs, clearly referencing the studies included	Pages 5,6	
Results			
**8** Reporting results	For each comparison and outcome, provide a description of the synthesised findings, and the certainty of the findings. Describe the result in language that is consistent with the question the synthesis addresses, and indicate which studies contribute to the synthesis	Pages 7-11, Table S3 and Table S4 in Online Supplementary Document	
Discussion			
**9** Limitations of the synthesis	Report the limitations of the synthesis methods used and/or the groupings used in the synthesis, and how these affect the conclusions that can be drawn in relation to the original review question	Page 15-16	

PRISMA=Preferred Reporting Items for Systematic Reviews and Meta-Analyses.

*If the information is not provided in the systematic review, give details of where this information is available (e.g., protocol, other published papers (provide citation details), or website (provide the URL)).
